# Nigrostriatal tau pathology in parkinsonism and Parkinson’s disease

**DOI:** 10.1093/brain/awad388

**Published:** 2023-11-25

**Authors:** Yaping Chu, Warren D Hirst, Howard J Federoff, Ashley S Harms, A Jon Stoessl, Jeffrey H Kordower

**Affiliations:** ASU-Banner Neurodegenerative Disease Research Center, Arizona State University, Tempe, AZ 85281, USA; Neurodegenerative Diseases Research Unit, Biogen, Cambridge, MA 02142, USA; Aligning Science Across Parkinson’s (ASAP) Collaborative Research Network, Chevy Chase, MD 20815, USA; Neurology, School of Medicine, Georgetown University Medical Center, Washington, DC 20007, USA; Department of Neurology, University of Alabama at Birmingham, Birmingham, AL 35294, USA; Aligning Science Across Parkinson’s (ASAP) Collaborative Research Network, Chevy Chase, MD 20815, USA; Pacific Parkinson’s Research Centre and Djavad Mowafaghian Centre for Brain Health, University of British Columbia, Vancouver, BC V6T 1Z3, Canada; ASU-Banner Neurodegenerative Disease Research Center, Arizona State University, Tempe, AZ 85281, USA; Aligning Science Across Parkinson’s (ASAP) Collaborative Research Network, Chevy Chase, MD 20815, USA

**Keywords:** tau, alpha-synuclein, dopaminergic neurodegeneration, parkinsonism, Parkinson’s disease

## Abstract

While Parkinson’s disease remains clinically defined by cardinal motor symptoms resulting from nigrostriatal degeneration, it is now appreciated that the disease commonly consists of multiple pathologies, but it is unclear where these co-pathologies occur early in disease and whether they are responsible for the nigrostriatal degeneration.

For the past number of years, we have been studying a well-characterized cohort of subjects with motor impairment that we have termed mild motor deficits. Motor deficits were determined on a modified and validated Unified Parkinson’s Disease Rating Scale III but were insufficient in degree to diagnose Parkinson’s disease. However, in our past studies, cases in this cohort had a selection bias, as both a clinical syndrome in between no motor deficits and Parkinson’s disease, plus nigral Lewy pathology as defined post-mortem, were required for inclusion. Therefore, in the current study, we only based inclusion on the presence of a clinical phenotype with mild motor impairment insufficient to diagnose Parkinson’s disease. Then, we divided this group further based upon whether or not subjects had a synucleinopathy in the nigrostriatal system.

Here we demonstrate that loss of nigral dopaminergic neurons, loss of putamenal dopaminergic innervation and loss of the tyrosine hydroxylase-phenotype in the substantia nigra and putamen occur equally in mild motor deficit groups with and without nigral alpha-synuclein aggregates. Indeed, the common feature of these two groups is that both have similar degrees of AT8 positive phosphorylated tau, a pathology not seen in the nigrostriatal system of age-matched controls. These findings were confirmed with early (tau Ser208 phosphorylation) and late (tau Ser396/Ser404 phosphorylation) tau markers. This suggests that the initiation of nigrostriatal dopaminergic neurodegeneration occurs independently of alpha-synuclein aggregation and can be tau mediated.

## Teaser

By comparing the brains of patients with mild motor deficits with and without nigral synucleinopathy and patients with sporadic Parkinson's disease, Chu *et al.* provide evidence that the initiation of nigrostriatal dopaminergic neurodegeneration occurs independently of alpha-synuclein aggregation and can be tau mediated.

See Espay and Lees (https://doi.org/10.1093/brain/awae002) for a scientific commentary on this article.

## Introduction

Nigral dopaminergic neurodegeneration and accumulation of aggregated proteins in Lewy bodies (LB) and Lewy neurites (LN) pathologically define Parkinson’s disease (PD). LB and LN, collectively referred to as Lewy pathology, are required for the post-mortem diagnosis of definite PD^1^ and are considered a precursor to neuronal degeneration.^2^ According to Braak staging,^3^ LB deposition follows a predictable sequence, progressing in a stereotypical pattern starting caudally from the lower brainstem and moving rostrally with involvement of the substantia nigra in Braak Stage 3. Alternatively, pathology may originate in the olfactory bulb. However, Braak PD Stages 1 and 2, occur ‘before’ Lewy body pathology occurs in the substantia nigra, and in those stages there are already reduced neuronal densities of tyrosine hydroxylase (TH) positive neurons and a higher percentage of TH-immunonegative melanin-laden neurons.^4^ This suggests that neurodegeneration and neuronal dysfunction precede alpha-synuclein (α-syn) positive Lewy pathology in the substantia nigra. Furthermore, Lewy pathology is not always detected in the substantia nigra of PD and parkinsonian brains,^4–8^ indicating that there is an early non-synuclein pathologic process involved in PD nigrostriatal degeneration, challenging the central pathogenic role of α-syn.

Tau is a normally-occurring protein that is subject to extensive post-translational modifications such as hyperphosphorylation, truncation and deglycosylation, resulting in insoluble, misfolded and aggregated protein isoforms. This may cause disruption in the microtubule network and impairment of axonal transport, eventually causing synaptic and neuronal degeneration.^9^ Tau inclusions have been found in nigral neurons by direct immunochemical studies of partially purified Lewy bodies and indirect immunohistochemical studies.^10^ In some studies, 50% of PD brains have tau inclusions.^11^ Furthermore, gait impairment in older people is associated with tau aggregation in substantia nigra.^12^ PD was not initially considered to be a typical tauopathy. However, several studies have demonstrated increasing evidence of tau pathology in PD brain.^11,13–16^ Genome-wide association studies (GWAS) of the sporadic form of PD^11,17^ have identified *MAPT,* encoding the microtubule-associated protein tau, as being associated with an increased risk of disease.^18^ Whether tau pathology precedes Lewy pathology and the role that it plays in nigrostriatal degeneration is an important question that has not been addressed to date.

We have studied a cohort of subjects who had minor motor deficits but a clinical syndrome insufficient for a PD diagnosis.^19^ We have termed this group mild motor deficits (MMD).^19–21^ To support this concept, we have found that subjects in this cohort display decreases in TH-immunoreactive (TH-ir) nigral neurons, decreases in putamenal TH innervation and decreases in dopaminergic phenotype relative to subjects with no motor deficits (NMD) but not to the degree that is seen in PD.^19^ However, subjects in the MMD group had two preselected defining criteria: (i) a clinical motor syndrome that was measurable but insufficient to be diagnosed with PD; and (ii) a synucleinopathy within the substantia nigra. To eliminate this bias resulting from all cases having synucleinopathy, we collected new cases where the only requirement was for subjects to have clinical evidence of MMD. Without bias, we assessed these cases in a blinded fashion and further categorized them as to whether a synucleinopathy was present (MMD-LB) or absent (MMD) in order to test the hypothesis that synucleinopathy was responsible for nigrostriatal degeneration. Indeed, for all our measures, MMD and MMD-LB were statistically identical. Rather strikingly, pathological tau was present in all MMD and MMD-LB cases and 22 of 24 PD cases and thus may be implicated in nigrostriatal degeneration and the development of motor PD.

## Materials and methods

### Subjects

We analysed brain tissues from older adults with NMD (*n* = 9), MMD (*n* = 28) and sporadic PD (*n* = 24). Subjects with MMD were further categorized based on the presence (MMD-LB, *n* = 17) or absence (MMD, *n* = 11) of nigral Lewy pathology (Table 1). Each subject signed an informed consent for clinical assessment prior to death and an anatomic gift act for donation of brain at the time of death. These subjects with NMD, MMD and MMD-LB were participants in the Religious Orders Study, a community-based cohort study of chronic conditions of ageing, who agreed to brain autopsy at the time of death and were examined by a neurologist or geriatrician at the Rush Alzheimer’s Disease Center. All adults with sporadic PD were diagnosed by movement disorder specialists in the department of Neurological Sciences at Rush University Medical Center. All cases were evaluated pathologically by a board-certified neuropathologist who confirmed that these subjects did not have pathologies that met diagnostic criteria for any other neurodegenerative disease. The Human Investigation Committee at Rush University Medical Center approved this study.

**Table 1:** Clinical and postmortem characteristics (mean ± standard deviation)

Measure	No Motor Deficit (NMD)	Minimal Motor Deficit with Absent nigral Lewy body (MMD)	Minimal Motor Deficit with nigral Lewy body (MMD-LB)	Parkinson’s disease (PD)
Case number	9	11	17	24
Age at death, years	81.33 ± 8.06	93.00 ± 3.10	90.41 ± 5.53	78.04 ± 8.69^##,$$^
Sex, male/female	4/5	3/8	9/8	13/11
Global parkinsonism, (0–100)	4.32 ± 2.05	25.37 ± 11.92**	16.34 ± 12.41*	38.64 ± 10.24***^,$$^
Parkinsonian gait score (0–100)	9.56 ± 11.48	56.96 ± 19.68***	34.27 ± 23.15*	56.07 ± 22.34***
Rigidity (0–100)	4.04 ± 8.93	16.36 ± 19.76	16.8 ± 22.54	38.33 ± 17.29***^,#,$$^
Tremor (0–100)	1.54 ± 4.63	2.47 ± 4.85	3.49 ± 5.56	8.07 ± 12.27
Bradykinesia (0–100)	2.18 ± 6.55	26.81 ± 16.05*	15.75 ± 14.47	52.10 ± 16.28***^,#,$$$^
Post-mortem interval, h	5.88 ± 2.45	10.24 ± 4.07	9.37 ± 6.37	5.99 ± 2.41

^*^ *P* < 0.05, **P < 0.01 and ***P < 0.001 compared with NMD.

^#^ *P* < 0.05 and ^##^*P <* 0.01 compared with MMD.

^$$^ *P* < 0.01 and ^$$$^*P <* 0.001 compared with MMD-LB.

### Tissue processing and post-mortem evaluation

At autopsy, the brains were removed from the calvarium and processed as described previously.^22,23^ Briefly, each brain was cut into 2 cm coronal slabs and then hemisected. The slabs were fixed in 4% paraformaldehyde for 5 days at 4°C. Twenty-four brain blocks were sampled from one side of the brain for pathologic diagnoses; the remaining brain slabs were cryoprotected in 0.1 M PBS (pH 7.4) containing 2% dimethyl sulfoxide, 10% glycerol for 2 days, followed by 2% dimethyl sulfoxide and 20% glycerol in PBS for at least 2 days prior to sectioning. The fixed slabs were then cut into 18 adjacent series of 40 μm thick sections on a freezing sliding microtome. All sections were collected and stored at −20°C in a cryoprotectant solution prior to processing.

A complete neuropathologic evaluation was performed.^12^ Dissection of diagnostic blocks included a hemisection of brain, including substantia nigra and striatum. When present, LBs were identified with haematoxylin and eosin (H&E) staining and further visualized with antibody staining for α-syn (details in the Supplementary material) from midfrontal, midtemporal, inferior parietal, anterior cingulate, entorhinal cortex and hippocampus, amygdala, basal ganglia and midbrain. McKeith criteria^24^ were modified to assess the categories of LB disease. Nigral neuronal loss was estimated from midbrain (details in the Supplementary material). Bielschowsky silver stain was used to visualize neurofibrillary tangles in the frontal, temporal, parietal, entorhinal cortex and hippocampus. Braak stages were based upon the distribution and severity of neurofibrillary tangle pathology. The neuritic plaque density was scored as recommended by the Consortium to Establish a Registry for Alzheimer’s Disease (CERAD). In addition to the evaluation by a board-certified neuropathologist, we performed an evaluation of Lewy pathology using α-syn immunohistochemistry, allowing for the segregation of MMD into MMD and MMD-LB groups, which was confirmed by the lead and senior authors.

### Immunohistochemistry

An immunoperoxidase labelling method^25^ was used to visualize phosphorylated α-syn using monoclonal phospho-S129 antibody (p-S129, 1:1000, Cat# ab51253, Abcam, RRID:AB_869973), TH expression using a monoclonal TH antibody (1:10 000, ImmunoStar; Cat# 22941, RRID: AB572268) and phosphorylated paired helical filament tau with phospho-Ser202+Thr205 using a monoclonal antibody (AT8, 1:1000; Cat# MN1020, Thermo Fisher Scientific, RRID:AB_223647),^26^ phospho-serine 202 (CP13, 1:1000, gift from Peter Davies, P. Davies Albert Einstein College of Medicine; New York; USA, Cat# CP13, RRID:AB_2314223) and phospho-serine396/serine404 (PHF-1, 1:1000, gift from Peter Davies, P. Davies Albert Einstein College of Medicine; New York; USA, Cat# PHF1, RRID:AB_2315150). One series of sections (1 of 18 series) was stained for each of the main antibodies (TH, AT8 and p-S129) and AT8/TH double-labelling^25^ and analysed using a stereological estimate.^27^ We randomly selected 3–4 cases in each group and three sections from each case for CP13, PHF-1, 3R-tau, 4R-tau and thioflavin staining. Qualitative analyses were performed on these immunolabelling procedures.

Endogenous peroxidase was quenched by 20 min incubation in 0.1 M sodium periodate, and background staining was blocked by 1 h incubation in a solution containing 2% bovine serum albumin and 5% normal horse serum or goat serum. Tissue sections were immunostained for primary antibody overnight at room temperature. After six washes, sections were sequentially incubated for 1 h in biotinylated horse anti-mouse IgG (for TH and AT8, Vector Laboratories, Cat# BA-2000, RRID:AB_2313581) or goat anti-rabbit IgG (for p-S129, Vector Laboratories, Cat# BA-1000, RRID:AB_2313606), followed by the Elite avidin-biotin complex (1:500; Vector Laboratories, Cat# PK-6100) for 75 min. The immunohistochemical reaction was completed with 0.05% 3,3′-diaminobenzidine (DAB) and 0.005% H_2_O_2_. Stained sections were mounted on gelatin-coated slides, dehydrated through graded alcohol, cleared in xylene and coverslipped with Cytoseal (Richard-Allan Scientific, Cat# 8310-16).

Immunohistochemical control experiments included omission of the primary antibodies (which control for the specificity of the staining procedure). The control sections were processed in a manner identical to that described above. All primary antibodies-delete control experiments resulted in the absence of specific staining.

A preadsorption control experiment for p-S129 antibody was also performed. Briefly, the p-S129 antibody was combined with a 5-fold (by mass) of p-S129 recombinant peptide (Abcam, Cat# ab188826) in Tris-buffered saline and incubated overnight at 4°C. The immune complexes with the antibody and blocking peptide were centrifuged at 10 000*g* for 20 min. The adsorbed peptide/antibody supernatant was then used in lieu of the primary antibody. This resulted in a total absence of staining (Supplementary Fig. 1A and B). To examine whether AT8 antibody cross-reacted with α-syn inclusions, the AT8 antibody was combined with p-S129 peptide. Immunohistochemistry revealed nigral AT8-immunopositive aggregates were the same with or without preadsorption of AT8 antibody/p-S129 peptide (Supplementary Fig.1C and D). Additionally, the staining patterns for TH^19,28^ and tau^29^ were similar to those reported previously, the latter of which is an important finding, because it demonstrates that the presence of AT8 stained tau did not cross-react with the p-S129 antibody. Stereology evaluated densities of TH immunoreactive neurons and AT8 and p-S129 immunoreactive aggregates and immunofluorescent double or triple labelling are presented in the Supplementary material.

### Data analyses

Clinical characteristics were compared across groups using a Kruskal–Wallis ANOVA and, where appropriate, followed by a Dunn’s multiple *post hoc* test. Neuronal number, aggregate number, particle densities and optical density measurements were compared across groups using one-way ANOVA followed by Tukey *post hoc* tests controlling for multiple comparisons (GraphPad Prism v4,RRID:SCR_002798, https://www.graphpad.com). The level of significance was set at 0.05 (two-tailed).

### Digital illustrations

Conventional light microscopic images were acquired using an Olympus microscope (BX61) attached to a Nikon digital camera (DXM1200) and stored as tiff files. Confocal images were exported from the Olympus laser-scanning microscope with Fluoview software (Olympus FV10-ASW v4.2b, RRID:SCR_014215, https://www.olympus-lifescience.com) and stored as tiff files. All figures were prepared using Photoshop 7.0 graphics software (Adobe Photoshop v7.0, RRID:SCR_014199, https://www.adobe.com/products/photoshop.html). Only minor adjustments of brightness were made.

## Results

### Parkinsonian signs in subjects with and without a clinical diagnosis of Parkinson’s disease

We analysed brain tissues from 9 older adults with NMD, 11 older adults with MMD, 17 older adults with MMD-LB and 24 adults with a clinical diagnosis of PD. The diagnoses of subjects with MMD, MMD-LB and PD were confirmed pathologically in each case, and there was no evidence of atypical Parkinsonism (e.g. progressive supranuclear palsy, corticobasal degeneration or multiple system atrophy), nor of other tauopathy (Alzheimer’s disease, frontotemporal disorders) in any subject. Demographics are provided in Table 1. NMD subjects displayed low global scores as well as low scores on individual signs of gait, bradykinesia, rigidity and tremor. In contrast, subjects with PD exhibited significantly higher scores on each of these measures. For the MMD and MMD-LB cases, the bradykinesia and parkinsonian gait were significantly greater compared with NMD cases. Other scores were intermediate, but no statistical differences between groups were observed. There was no difference in tremor score among groups. Detailed clinical examinations from the individuals are presented in Supplementary Tables 1–4.

### Qualitative and quantitative observations of nigral and putamenal phosphorylated α-syn immunoreactivity

**Figure 1:**
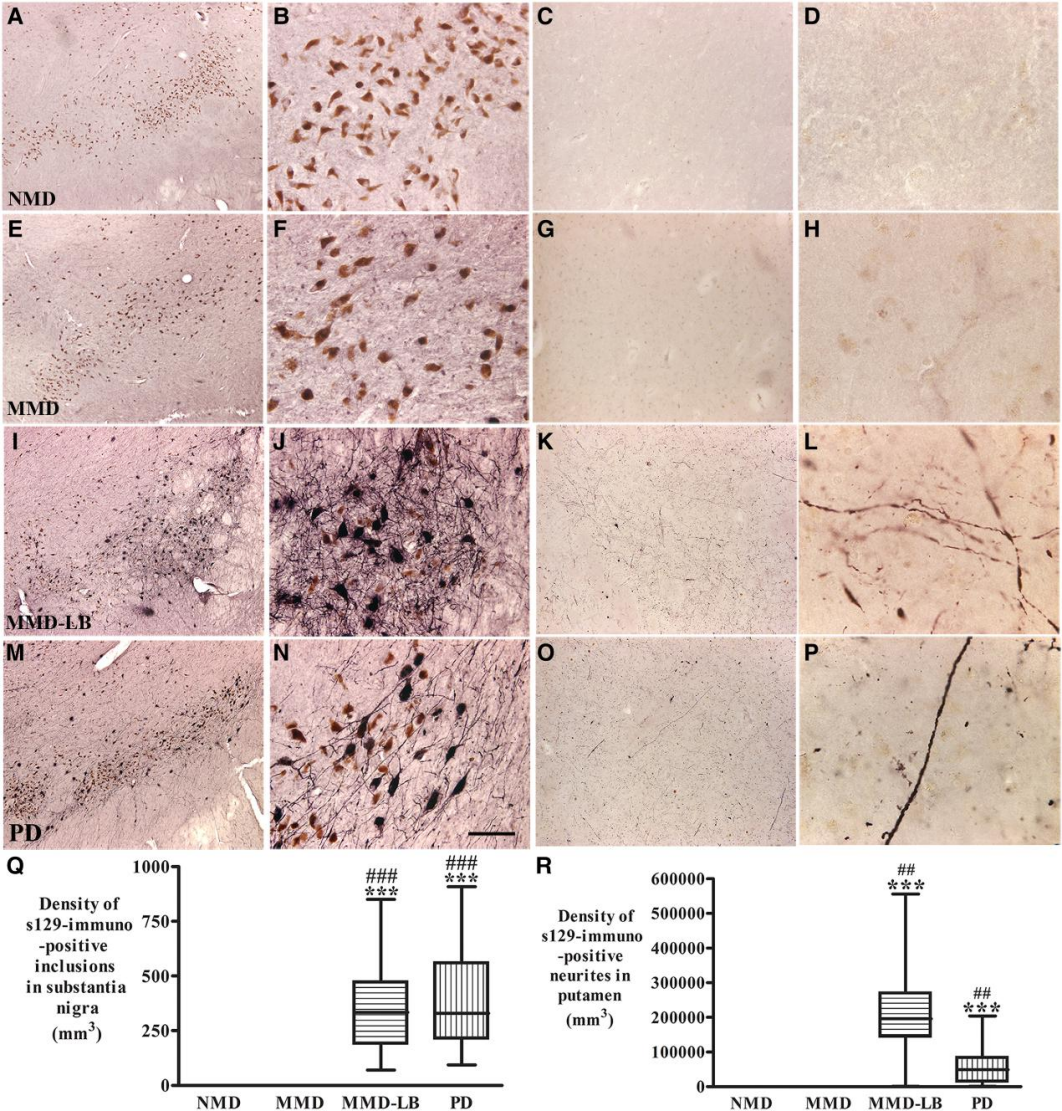
**Qualitative and quantitative evaluation of phosphorylated α-synuclein aggregates in substantia nigra and putamen.** Photomicrographs of the mid-substantia nigra (*left two columns*) and putamen (*right two columns*) from no motor deficit (NMD; **A**–**D**), minimal motor deficits (MMD; **E**–**H**), minimal motor deficits with nigral Lewy body (MMD-LB; **I**–**L**) and Parkinson’s disease (PD; **M**–**P**) show phospho-S129 α-syn (p-S129) staining patterns. p-S129 immunoreactivity was undetectable in the subjects with NMD (**A**–**D**) and MMD (**E**–**H**). In contrast, p-S129-immunoreactive nigral neurons were observed in subjects with MMD-LB (**I** and **J**) and patients with PD (**M** and **N**) and p-S129-immunoreactive neurites in putamen were detectable in both subjects with MMD-LB (**K** and **L**) and PD (**O** and **P**). Scale bar in **N** = 100 µm in **B**, **F** and **J**, 500 µm in **A**, **E**, **I** and **M**; 200 µm in **C**, **G**, **K** and **O**, 20 µm in **D**, **H**, **L** and **P**. Stereological analyses revealed that no p-S129-positive aggregate in substantia nigra (**Q**) and putamen (**R**) was counted in NMD and MMD groups. However, there was a significantly higher densities of p-S129 labelled aggregates in MMD-LB and PD groups. ***P < 0.001 compared with NMD. ^##^*P* < 0.01 and ^###^*P* < 0.001 compared with MMD.

To verify that subjects from the MMD group had no nigral Lewy pathology undiagnosed by the neuropathologists, we examined the phosphorylated α-syn labelling in substantia nigra and putamen in all participants. Qualitative and quantitative analyses revealed that phosphorylated α-syn aggregates were widely distributed through substantia nigra in MMD-LB (355.59 ± 210.01/mm^3^) and PD (398.69 ± 227.15/mm^3^) groups but not in NMD and MMD groups (Fig. 1 and Supplementary Figs 2–5). The phosphorylated α-syn neurites were observed throughout putamen in MMD-LB (206 927.3 ± 150 917.3/mm^3^) and PD (55 659.97 ± 56 892.64/mm^3^) groups but not in NMD and MMD groups (Fig. 1). These data further demonstrate that MMD subjects with motor deficits did not display nigral and putamenal α-syn pathology.

### Characteristics of tyrosine hydroxylase expression in the nigrostriatal system

**Figure 2:**
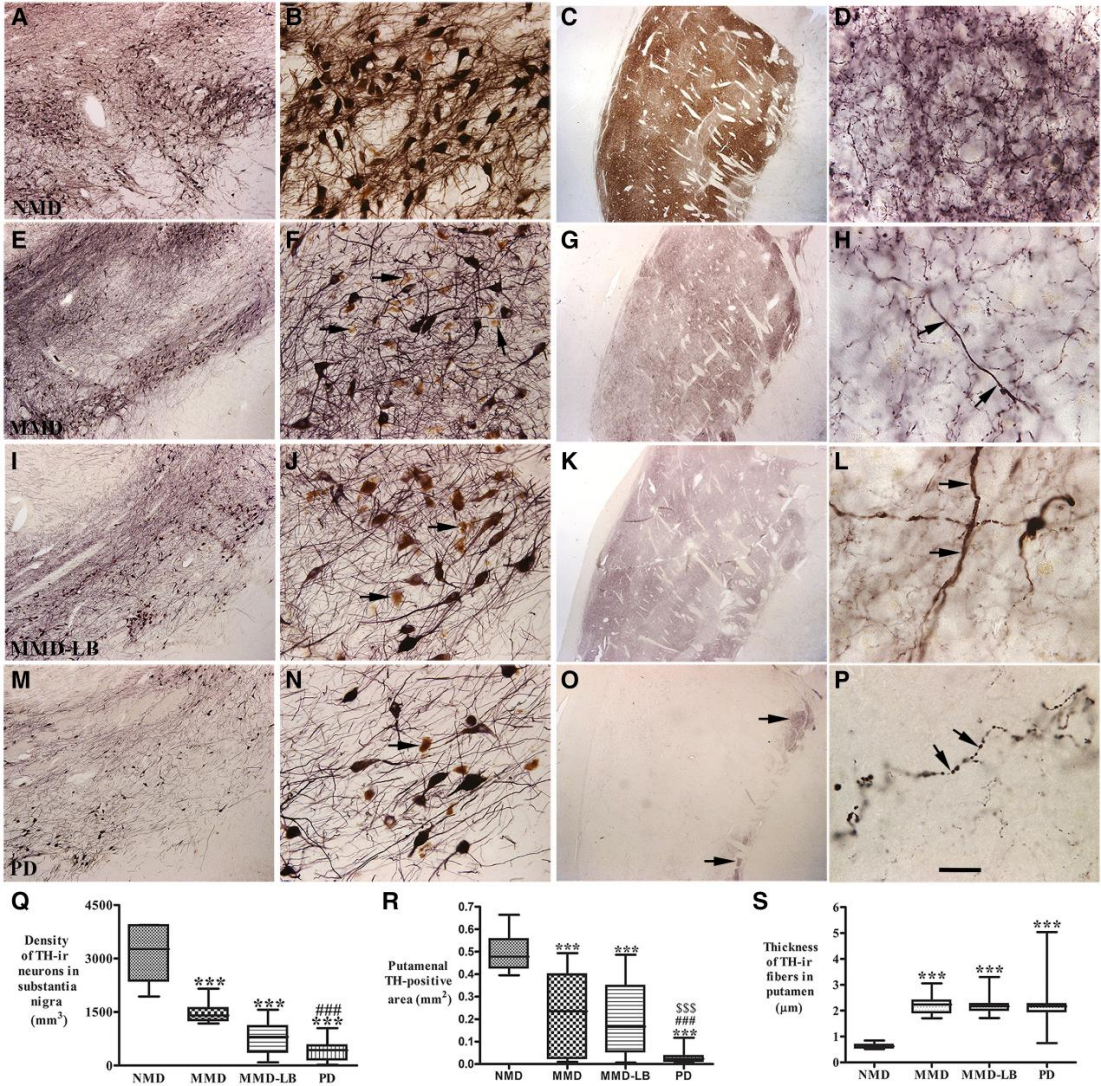
**Qualitative and quantitative evaluation for nigral and putamenal tyrosine hydroxylase expression.** Photomicrographs of substantia nigra (*left two columns*) and putamen (*right two columns*) from no motor deficit (NMD; **A**–**D**), minimal motor deficits (MMD; **E**–**H**), minimal motor deficits with Lewy body pathology (MMD-LB; **I**–**L**) and Parkinson’s disease (PD; **M**–**P**) illustrate tyrosine hydroxylase (TH) immunoreactivity. Subject from the NMD group showed intense TH-immunoreactive (TH-ir) somata with extensive local plexus of TH-ir processes (**A** and **B**) in substantia nigra and dense TH-ir fibres throughout putamen (**C** and **D**). Nigral TH immunoreactivity was reduced in MMD (**E** and **F**) and MMD-LB subjects (**I** and **J**) compared with NMD (**A** and **B**) and some remaining nigral melanized neurons exhibited no detectable TH immunoreactivity (arrows; **F** and **J**). Putamenal TH immunoreactivities were reduced in MMD (**G** and **H**) and MMD-LB (**K** and **L**) and some of remaining TH-ir fibres displayed swollen varicosities (arrows; **H** and **L**). PD cases displayed severe reduction of TH immunoreactivity in substantia nigra (**M** and **N**) and undetectable TH immunoreactivity in major putamen (**O** and **P**), except the ventromedial putamen near globus pallidus (arrows; **O**) and few remaining TH immunoreactive fibres exhibited swollen segments (arrows; **P**). Scale bar in **P** = 20 µm in **D**, **H**, **L**, 2.0 mm in **C**, **G**, **K** and **O** , 100 µm in **B**, **F**, **J** and **N**, 500 µm in **A**, **E**, **I** and **M**. (**Q**) Stereological analyses revealed that the density of TH-positive neurons was gradually reduced from MMD, MMD-LB to PD relative to the NMD group. Particle analyses (**R**) demonstrated that the TH-immunoreactive area/mm^2^ of putamen was accordingly reduced from MMD, MMD-LB to PD relative to the NMD group. (**S**) The thickness of remaining TH-ir fibres were larger in MMD, MMD-LB and PD relative to NMD. ***P < 0.001 compared with NMD, ^###^*P* < 0.001 compared with MMD and ^$$$^*P* < 0.001 compared with MMD-LB.

Tyrosine hydroxylase expression in subtantia nigra. Subjects from the NMD group had a high density of TH-ir somata and an intricate local plexus of TH-ir processes within the substantia nigra (Fig. 2A and B). A few NM-laden nigral neurons were TH-immunonegative. MMD subjects showed a clear reduction in TH-ir neurons (Fig. 2E). Many NM-laden nigral neurons were TH-immunonegative and the TH-ir neurons displayed less extensive processes (Fig. 2F). MMD-LB subjects (Fig. 2I) also displayed obvious reductions of TH-immunoreactivity, and qualitatively this reduction appeared identical to the MMD cases (Fig. 2E). As with the MMD cases, many NM-laden neurons in the MMD-LB cases displayed TH-immunonegative perikarya and decreased TH-ir arborization (Fig. 2J). In PD subjects, both TH-ir somata and dendrites in substantia nigra (Fig. 2M and N) were severely reduced to a degree greater than that seen in subjects with MMD and MMD-LB. Additional images for TH-immunostaining are presented in Supplementary Figs 2–5.

Stereological analyses confirmed that the densities of TH-ir neurons were decreased in PD cases (430.14 ± 283.91/mm^3^) relative to NMD subjects (3168.48 ± 770.76/mm^3^). There were similar, but smaller reductions in TH-ir nigral neurons in the MMD (1479.38 ± 282.70/mm^3^) and MMD-LB (842.53 ± 435.76/mm^3^) subjects relative to the NMD group. Relative to the NMD group, the reduction of nigral TH-ir neurons was 53.30% in MMD, 73.29% in MMD-LB and 85.78% in PD. An ANOVA revealed a statistically significant difference across these groups (Fig. 2Q; *P* < 0.0001). *Post hoc* analyses demonstrated a significant reduction of TH-labelled neurons in MMD (*P* < 0.001), MMD-LB (*P* < 0.001) and PD (*P* < 0.001) compared with the NMD group, and between the MMD and PD groups (*P* < 0.001). Critically, there was no significant difference between MMD and MMD-LB (*P* > 0.05) groups, indicating that Lewy pathology does not affect the loss of TH-ir neurons (in MMD) and suggests that factors other than Lewy pathology may be responsible for nigral cell loss.

#### Tyrosine hydroxylase-innervation of the putamen

TH-labelled dopaminergic terminals from NMD subjects were distributed in a mosaic pattern of low- and high TH-ir zones (Fig. 2C). Dense fine TH-ir fibres were distributed throughout the grey matter of putamen, consisting of a fine mesh of fibre (Fig. 2D). In contrast, TH-labelling from MMD subjects (Fig. 2G and H) was remarkably decreased compared with NMD controls (Fig. 1C and D). The TH-ir fine fibres were markedly reduced (Fig. 1H), although the light TH-labelling mosaic pattern remained (Fig. 2G). The TH-ir pattern from MMD-LB (Fig. 2K) and MMD (Fig. 2G) appeared similar. In both MMD groups, TH-ir fine fibres were diminished relative to NMD subjects and remaining putamenal fibres often displayed an abnormal morphology characterized by swollen varicosities (Fig. 2H and L). In each of the PD cases, TH-ir fine fibres were barely detectable in the putamen (Fig. 2O and P), and the few remaining thick fibres displayed swollen varicosities and varicose segments (Fig. 2P). A few fine TH-ir fibres were observed in the more ventromedial putamen proximal to the globus pallidus (Fig. 2O).

Measurement of TH-ir area supported the qualitative observations revealing that relative to the NMD group, putamenal TH-ir areas were reduced 56.75% in MMD, 60.94% in MMD-LB and 93.48% in PD. An ANOVA revealed a statistically significant difference across these groups (Fig. 2R; *P* < 0.0001). *Post hoc* analyses demonstrated a significant reduction of putamenal TH-ir areas in subjects with MMD (*P* < 0.001), MMD-LB (*P* < 0.001) and PD (*P* < 0.001) compared with the NMD group, between the MMD and PD groups (*P* < 0.001) and between the MMD-LB and PD groups (*P* < 0.001). Critically, there was no difference between the MMD and MMD-LB groups (*P* > 0.05), supporting the concept that α-syn pathology is not necessary for the loss of TH-innervation and suggests that factors other than Lewy pathology are responsible for putamenal loss of dopaminergic innervation.

From the morphologic analysis, putamenal thick TH-ir fibres were observed in subjects with MMD, MMD-LB and PD compared with the NMD group. To determine whether there was a difference among groups, the thickness of TH-ir fibres in putamen was measured for all participants using Feret’s diameter. These measurements revealed that remaining thick TH-ir fibres were larger in MMD (2.20 ± 0.11 µm), MMD-LB (2.19 ± 0.08 µm) and PD (2.24 ± 0.16 µm) compared with NMD (0.62 ± 0.02 µm). An ANOVA revealed a statistically significant difference across these groups (Fig. 2S; *P* < 0.0001). *Post hoc* analyses demonstrated a significantly greater putamenal TH-ir fibres in subjects with MMD (*P* < 0.001), MMD-LB (*P* < 0.001) and PD (*P* < 0.001) compared with the NMD group, but there was no difference among MMD, MMD-LB and PD groups (*P* > 0.05) These results reveal that, following the comprehensive loss of thin TH-ir putamenal fibres, some thick TH-ir fibres remained in subjects with MMD, MMD-LB and PD.

### Morphological characteristics of phosphorylated tau associated with the substantia nigra and putamen

**Figure 3:**
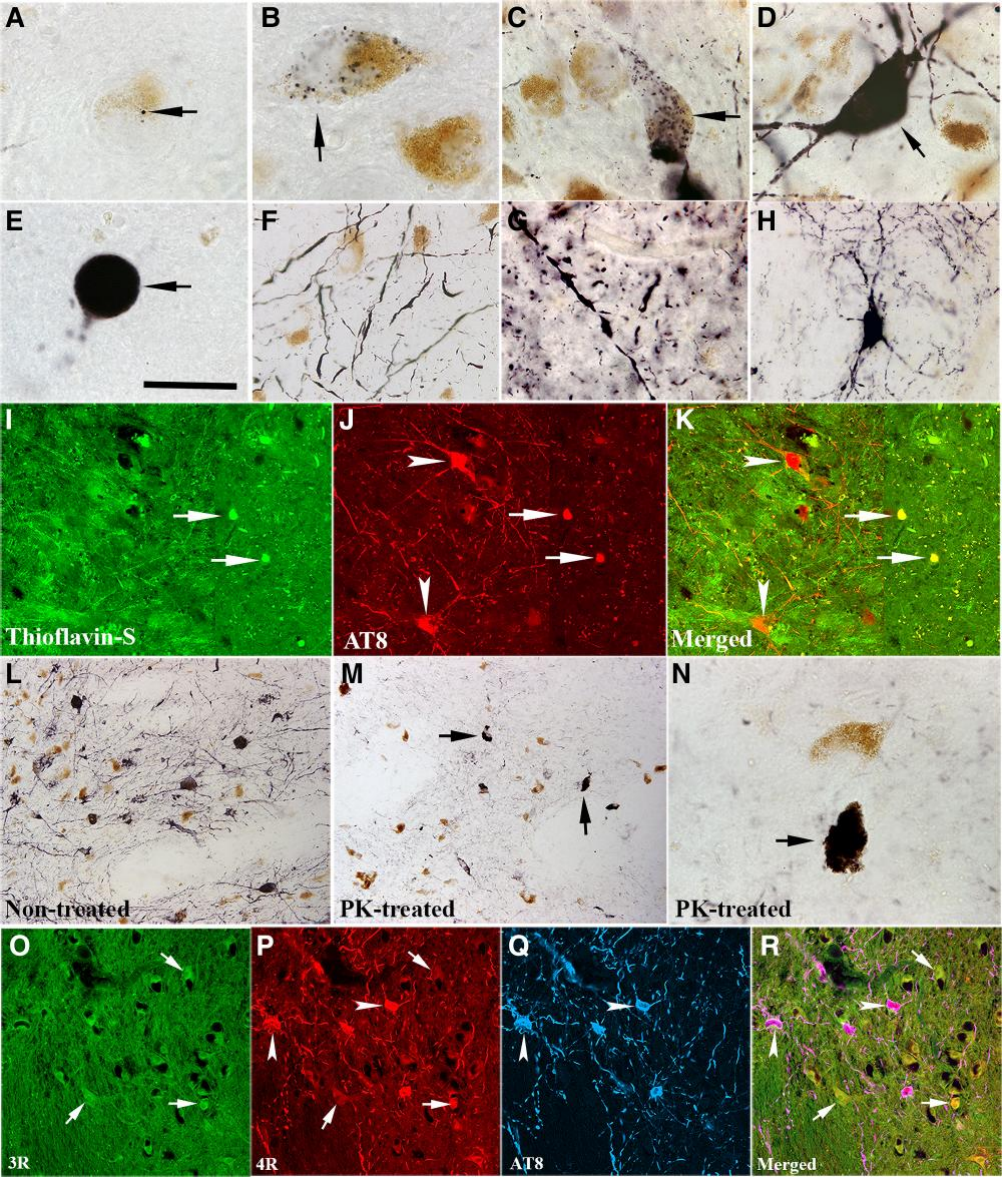
**Morphologic features of tau aggregation in nigrostriatal system.** Photomicrographs of substantia nigra (**A**–**F**) and putamen (**G** and **H**) show the shapes of AT8-immunoreactive (AT8-ir) aggregates. AT8-ir punctate granule was seeded (black, arrow; **A**) and mixed (black; arrow; **B**) into neuromelanin (NM; brown). AT8-ir clumpy granules were accumulated within perikarya (arrows; **C**) or filled with whole neuron including somata and main processes (arrow; **D**) that neurons displayed NM loss. AT8-ir spherical aggregates, like a Lewy body (arrow; **E**) with cytoplasm and processes disappear. AT8-ir fragmental threads (**F**) were distributed in substantia nigra. AT8-ir varicosities and punctuated boutons dispersed into putamen (**G**). Few AT8-ir putamenal neuron displayed dark stained somata with abundant processes (**H**). Double-labelling revealed the dense phosphorylated tau accumulations (AT8, red, arrows; **J**) were thioflavin-S positive (green, arrows; **I**) but the granules seen with phosphorylated tau were thioflavin negative (arrowheads; **I**–**K**). Phosphorylated tau immunolabelling (**L**) observed from the section without proteinase K (PK) treatment was basically eliminated following PK treated section (**M** and **N**). However, the dense tau accumulations (arrows; **M** and **N**) were resistant to PK treatment. Fluorescent triple-labelling (**O**–**R**) showed that both 3R (green, arrows; **O**) and 4R (red, arrows and arrowheads; **P**) tau isoforms existed in nigral melanized neurons. 4R isoform in same melanized neurons displayed stronger intense staining than 3R and co-localized with phosphorylated tau marker AT8 (blue, arrowheads; **P**–**R**). Scale bar in **E** = 20 µm in **A**–**H** and **N**, 100 µm in **I**–**K**, 120 µm in **L**, **M** and **O**–**R**.

Since α-syn pathology does not appear to be necessary for nigrostriatal degeneration in subjects with MMD, we examined the potential for tau to mediate this process, since tau has been shown previously to be a co-pathology in PD,^14,30^ and knockout of tau in preclinical models attenuates and delays neurodegeneration associated with α-syn.^31^ We examined tau pathology in all participants in this study. Phosphorylated tau aggregates were observed in the nigrostriatal system in all MMD and MMD-LB subjects and 22 of 24 PD subjects. Immunohistochemistry with AT8 antibody revealed multiple patterns of AT8-ir aggregates within the substantia nigra. Some nigral neurons had small AT8-labelled punctate granules (Fig. 3A and B) in highly melanized neurons. The distribution of neuromelanin (NM) in cells with and without AT8-labelled punctate granules appeared similar. As phosphorylated tau accumulates, AT8-labelled punctate granules fill the perikarya (Fig. 3C). Under certain circumstances, the phosphorylated tau filled the whole cell including perikarya and proximal processes (Fig. 3D) and the NM was not visible. Finally, the phosphorylated tau was seen to be compressed into a spherical shape (Fig. 3E). AT8-labelled processes displayed swollen threads and segments distributed through the substantia nigra (Fig. 3F).

In the putamen, AT8-labelled products exhibited swollen varicosities, segments and punctate boutons (Fig. 3G). The swollen varicosities present as beads-on-a-string-like axonal swellings. The punctate boutons appeared as granules distributed into putamenal grey matter. Fewer AT-8 labelled cells (Fig. 3H) displayed neuronal perikarya with oval or triangular shape and extensive local processes.

Double staining revealed that the dense tau aggregates were often thioflavin S-positive, but dispersed tau granules in perikarya and processes were not (Fig. 3I–K). Proteinase K digestion was used to further determine whether the AT8 labelled tau was soluble (non-aggregated) or insoluble (inclusions). In this experiment, proteinase K digested the granular tau structures but not the dense tau accumulation (Fig. 3L–N). These observations indicate that tau pathology progresses in the nigrostriatal system during PD development with apparent transition into aggregates that are largely resistant to protein K digestion.

**Figure 4:**
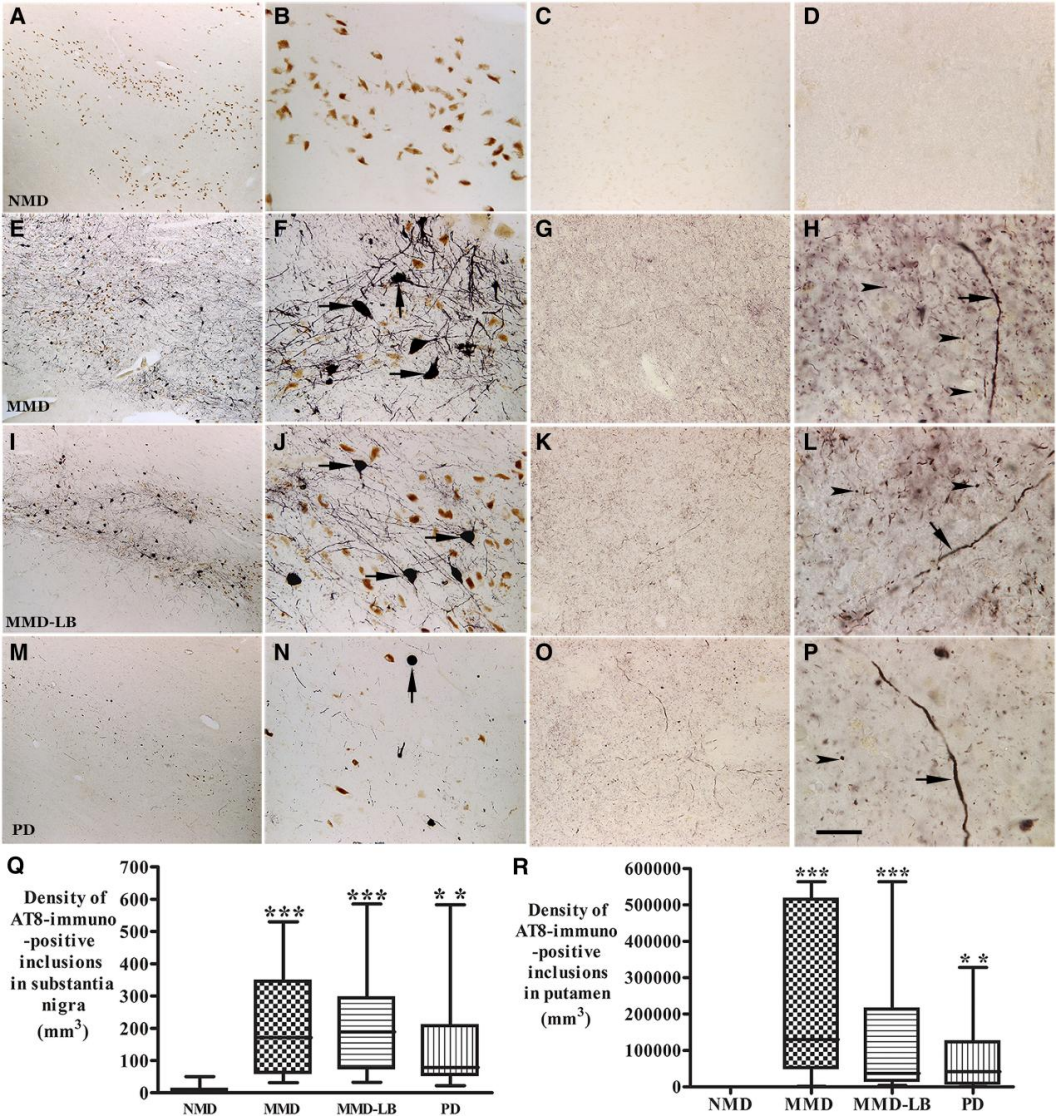
**Qualitative and quantitative evaluation of tau aggregates in substantia nigra and putamen.** Photomicrographs of the mid-substantia nigra (*left two columns*) and putamen (*right two columns*) from no motor deficit (NMD; **A**–**D**), minimal motor deficits (MMD; **E**–**H**), minimal motor deficits with nigral Lewy body (MMD-LB; **I**–**L**) and Parkinson’s disease (PD; **M**–**P**) show AT8-immunoreactive (AT8-ir) patterns. AT8 immunoreactivity was not detected in NMD group (**A**–**D**). In contrast, numerous AT8-ir neurons were distributed throughout substantia nigra (**E** and **I**) and displayed dark somata with abundant processes in subjects with MMD (arrows; **F**) and MMD-LB (arrows; **J**). AT8-ir intensities in putamen were higher in MMD (**G**) and MMD-LB (**K**) and displayed punctuated dots (arrowheads; **H** and **L**) and the same expanded fibre (arrow; **H** and **L**). The AT8-ir aggregates with limited processes (arrow; **N**) in substantia nigra (**M**) and relative less AT8-ir punctuated dots (arrowhead; **O** and **P**) in putamen were observed in PD. Scale bar in **P** = 20 µm in **D**, **H**, **L**, 100 µm in **B**, **C**, **F**, **G**, **J**, **K**, **N** and **O**, 500 µm in **A**, **E**, **I** and **M**. (**Q**) Stereological analyses revealed that the density of AT8-ir aggregates in substantia nigra and (**R**) the density of AT8-ir dots and threads in putamen were significant higher in MMD, MMD-LB and PD relative to NMD group. **P < 0.01 and ***P < 0.001 compared with NMD.

The adult human brain expresses multiple main tau isoforms, which can be categorized as 3R or 4R tau based on whether they contain three or four carboxy-terminal repeat domains.^32^ 3R and 4R tau is more common in Alzheimer’s disease, while 4R tau is seen in diseases such as progressive supranuclear palsy.^33^ To determine which isoform exists in the substantia nigra, the 3R and 4R isoforms were examined and co-localized with AT8-labelled phosphorylated tau. Fluorescent labelling revealed that both 3R (Fig. 3O) and 4R (Fig. 3P) tau isoforms were distributed in nigral NM-laden neurons. The intensity of 4R tau immunostaining was stronger than 3R tau. To further confirm these results, an immunoperoxidase method was used to examine 3R and 4R tau expression in nigral sections from age-matched control (NMD) and PD cases. As a staining control, we compared these staining patterns with similarly stained sections obtained from subjects with Alzheimer’s disease and age-matched control brains. Immunohistochemistry revealed that both 3R and 4R tau labelled nigral and cortical neurons showed similar intensity in control brains. In contrast, light 3R and intense 4R labelling were observed in nigral neurons in PD and in temporal cortical neurons in Alzheimer’s disease (Supplementary Fig. 6).

### Qualitative and quantitative observations of phosphorylated tau in nigrostriatal system

Our previous study^19^ indicated that the density of phosphorylated α-syn aggregates in subjects with MMD-LB was similar to PD. We now examined the distribution of tau inclusions from subjects with minimal motor deficits and clinically diagnosed PD. In this regard, AT8-labelled aggregates in substantia nigra and neuropil threads in putamen were examined and quantified in all subjects.

AT8-labelled nigral neurons displayed dark perikarya with abundant processes in subjects with MMD (Fig. 4E and F) and MMD-LB (Fig. 4I and J). In PD, AT8 stained spherical inclusions like somal Lewy bodies with fewer processes (Fig. 3N). AT8-immunoreactivity was undetectable in the NMD group (Fig. 4A and B). Additional images of AT8-immunolabelling are presented in Supplementary Figs 2–5. The density of AT8-labelled aggregates was variable from case to case. Stereological analyses showed that the density of nigral AT8-labelled aggregates was higher in subjects with MMD (223.78 ± 169.61/mm^3^) and MMD-LB (228.4 ± 196.57/mm^3^), medium in the PD (151.52 ± 165.75/mm^3^) and much lower in NMD (8.07 ± 17.54/mm^3^). An ANOVA revealed a statistically significant difference across these groups (Fig. 4Q; *P* < 0.001). *Post hoc* analyses demonstrated a significant higher density of AT8-labelled aggregates in MMD (*P* < 0.001), MMD-LB (*P* < 0.001) and PD (*P* < 0.01) compared with the NMD group. There was no difference between the MMD and MMD-LB (*P* > 0.05), between the MMD and PD (*P* > 0.05) or the MMD-LB and PD (*P* > 0.05) groups.

Tau phosphorylation was further investigated using two other commonly employed phosphorylated-tau antibodies, CP13 (for visualizing pretangles and mature-tangles)^34^ and PHF-1 (for visualizing mature-tangles).^35^ Immunohistochemistry revealed that both CP13 and PHF-1 labelled tau were widely distributed in substantia nigra in subjects with MMD, MMD-LB and PD but not in the NMD subjects (Supplementary Figs 7 and 8). There were more CP13 and PHF-1 labelled aggregates in subjects with MMD and MMD-LB than PD. The pattern of CP13 and PHF-1 labelled tau in substantia nigra was similar to AT8 staining, revealing that multiple phosphorylation sites contribute to tau aggregation in nigral dopaminergic neurodegeneration.

Within the putamen, AT8 immunoreactivity was virtually undetectable in NMD subjects (Fig. 4C and D). Different intensities of AT8 immunoreactivities were observed in the putamen of each subject with MMD, MMD-LB or PD. Both MMD and MMD-LB subjects had intense AT8-labelled products that were characterized by densely stained puncta, boutons, segments and swollen varicosities (Fig. 4G, H, K and L). The density of AT8-labelled punctuated boutons in the PD group (Fig. 4O and P) was relatively lower than that seen in the MMD and MMD-LB groups (Fig. 4H and L). Quantitative analyses showed that the density of nigral AT8-labelled aggregates was maximal in subjects with MMD (239 891.2 ± 223 426.5/mm^3^), high in MMD-LB (127 876.3 ± 174 805.8/mm^3^), intermediate in PD (78 948.9 ± 99 519.2/mm^3^) and much lower in NMD (40.37 ± 114.19/mm^3^). An ANOVA revealed a statistically significant difference across these groups (Fig. 4R; *P* < 0.0001). *Post hoc* analyses demonstrated a significantly higher density of AT8-labelling in MMD (*P* < 0.001), MMD-LB (*P* < 0.001) and PD (*P* < 0.01) compared with the NMD group, but there was no difference among the MMD, MMD-LB and PD groups (*P* > 0.05). These data indicate that tau pathology is present in the development of nigrostriatal degeneration, especially during the early stages and in cases in which Lewy pathology does not exist.

### Co-localization analysis of tau and α-synuclein inclusion in the nigrostriatal system

**Figure 5:**
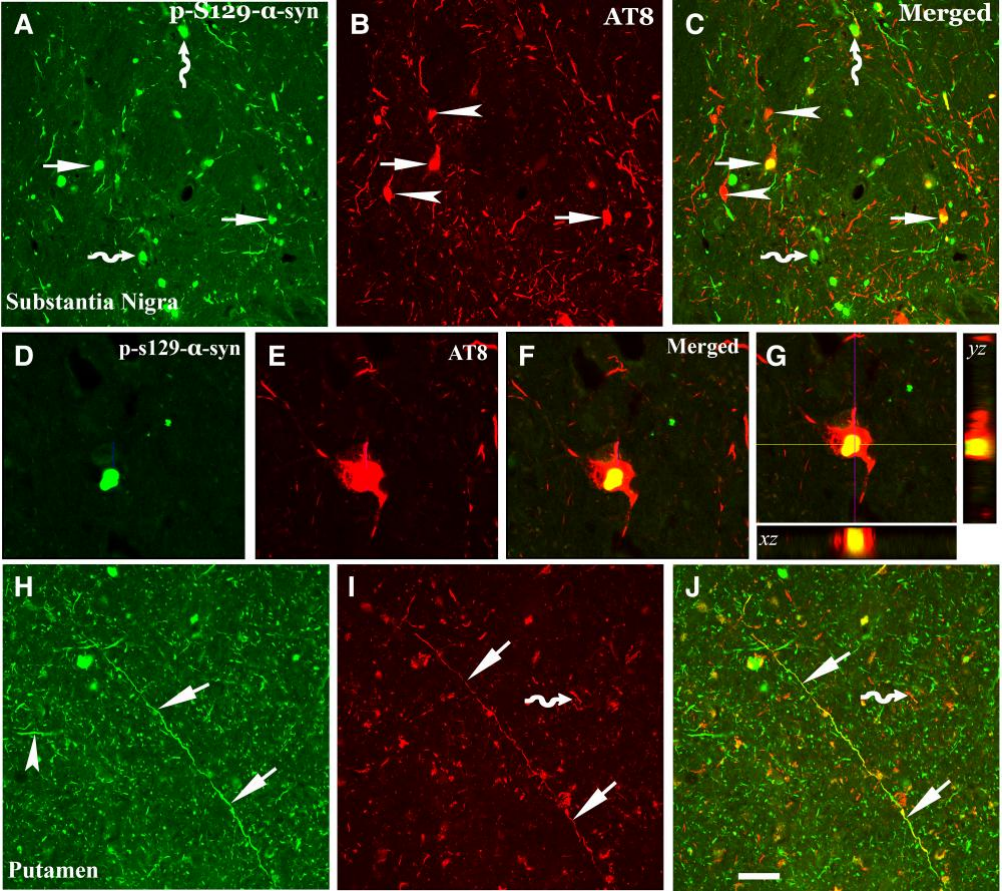
**Co-localization analyses of phosphorylated tau (AT8) and α**–**syn (p**–**S129**–**α**–**syn) in nigrostriatal system.** Confocal microscopic images of substantia nigra (**A**–**G**) and putamen (**H**–**J**) illustrated p-S129-α-syn (green; **A**, **D** and **H**), AT8 (red; **B**, **E** and **I**) and co-localization of AT8 and p-S129 (merged; **C**, **F**, **G** and **J**). There were three populations of immunofluorescence-labelling aggregates: p-S129-α-syn and AT8 double-labelling (arrows; **A**–**C**), AT8 single-labelling (arrowhead; **B** and **C**) or p-S129-α-syn single-labelling (curved arrow; **A** and **C**). The p-S129-α-syn-labelled aggregate (**D**) deposited within AT8-labelled perikarya (**E** and **F**). 3D reconstruction of confocal image further illustrated the co-localization of labelled p-S129-α-syn and AT8 (**G**): the large panel represents a cross section of the cell layer; the horizontal (yellow) and vertical (pink) lines through them denote the planes of the adjoining *xz* and *yz* sections, respectively. *Bottom* and *right*: The *xz* and *yz* cross sections were obtained from the combined serial optical sections of these cell layers using Olympus Confocal Fluoroview software. The 3D reconstruction analyses revealed that p-S129-α-syn were co-localized with AT8 (yellow). In putamen p-S129-α-syn (arrowhead; **H**) and AT8-labelled threads and dots (curved arrow; **I**) were not co-localized, but the longer fibre with AT8 labelling was p-S129-α-syn immunopositive (arrows; **H**–**J**). Scale bar in **J** = 50 μm in **H**–**J**, 25μm in **D**–**G**, 100μm in **A**–**C**.

The above noted findings of tau accumulation in the nigrostriatal system of subjects with MMD-LB and PD led us to hypothesize that tau and α-syn aggregates might co-exist in dopaminergic neurodegeneration in these populations. To address this, both tau and α-syn aggregation were examined using double-labelling with AT8 and p-S129-α-syn antibodies. Co-localization analyses demonstrated three neuronal populations in the substantia nigra: (i) neurons that co-localize AT8 and p-S129-α-syn (Fig. 5A–C); (ii) neurons with AT8-labelled inclusions alone (Fig. 5B and C) or (iii) p-S129-α-syn labelled aggregates alone (Fig. 5A and C). Co-localization analyses further revealed that the p-S129-α-syn inclusion deposited into neurons with AT8-staining somata and processes (Fig. 5D–G). However, only 5–8% of AT8-labelled aggregates co-localized with p-S129-α-syn within nigral neurons, as the majority of AT8 staining was not co-localized with α-syn. To further confirm these observations, an α-syn antibody that recognizes full length human wild-type α-syn was used to co-label with AT8. Double-labelling revealed that few AT8 aggregates were co-localized with α-syn, but most AT8 aggregates deposited independently in melanized neurons (Supplementary Fig. 9). These findings support the observations from ser-129 and AT8 double labelling. These antibody labelled α-syn-positive fibres and puncta were reduced in substantia nigra of MMD, MMD-LB and PD compared with NMD.

In the putamen, longer neuropil threads, labelled by AT8 were also p-S129 immunopositive (Fig. 5H–J) but short segments and puncta were not seen in subjects with MMD-LB and PD. These data are consistent with the notion that tau drives nigrostriatal degeneration.

### Down-regulation of dopaminergic markers in nigral neurons with tau aggregates

**Figure 6:**
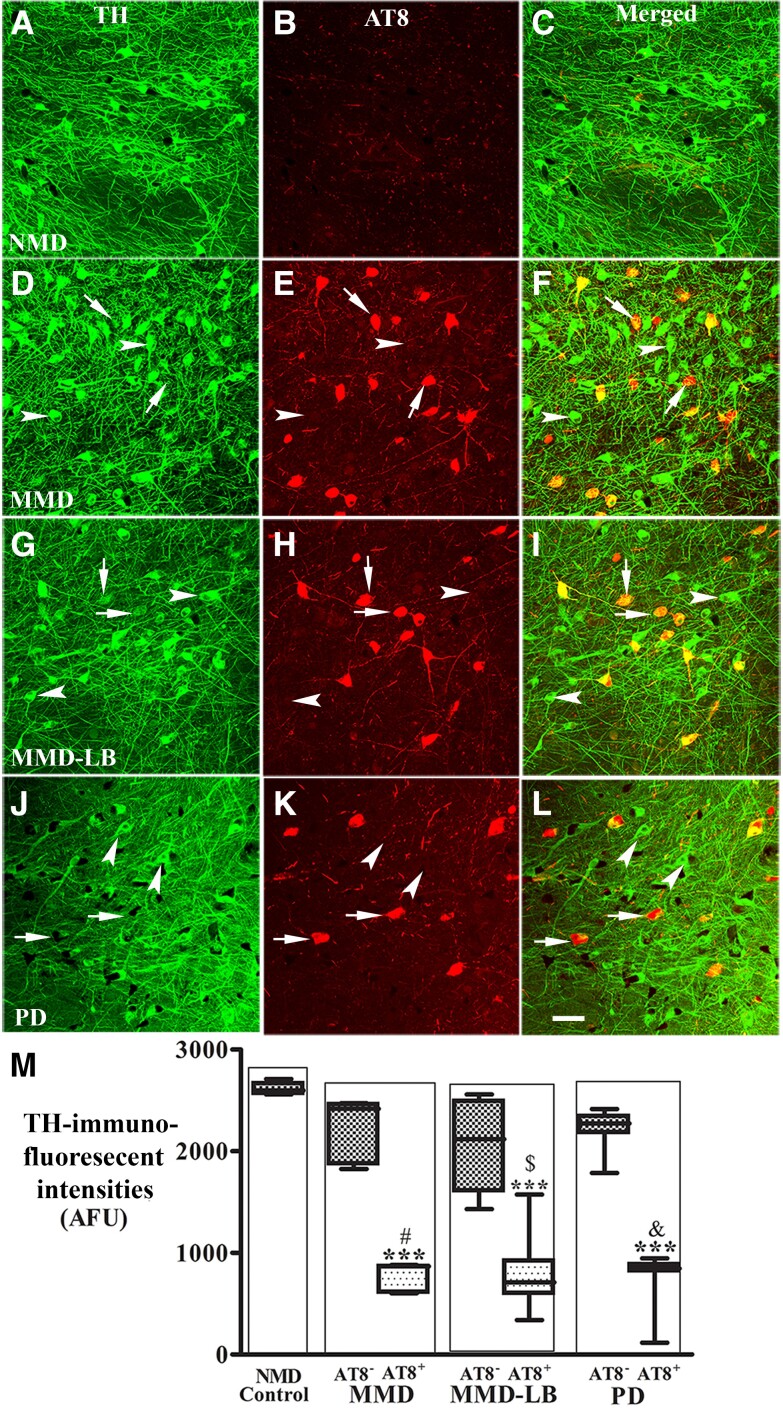
**Reduction of tyrosine hydroxylase levels in nigral neurons with AT8-immunorepressive aggregates.** Confocal microscopic images of substantia nigra from no motor deficit (NMD; **A**–**C**), minimal motor deficits (MMD; **D**–**F**), minimal motor deficits with nigral Lewy body (MMD-LB; **G**–**I**) and Parkinson’s disease (PD; **J**–**L**) illustrated immunostaining for tyrosine hydroxylase (TH; green; **A**, **D**, **G** and **J**), AT8 (red; **B**, **E**, **H** and **K**) and co-localization of TH and AT8 (merged; **C**, **F**, **I** and **L**). Note that TH immunofluorescent intensity was extensively reduced in the neurons with tau aggregates (arrows; **D**–**L**) but not in the neurons without tau aggregates (arrowheads; **D**–**L**). Scale bar in **L** = 100 µm (applies to all panels). Measurements of immunofluorescent intensities (**M**) further revealed that TH expression was significantly reduced in the neurons with tau aggregates (AT8^+^) but not in the neurons without tau aggregate (AT8^−^). ***P < 0.001 related to NMD control, ^#^*P* < 0.05 related to AT8 immunonegative neurons in MMD, ^$^*P* < 0.05 related to AT8 immunonegative neurons in MMD-LB and ^&^*P* < 0.05 related to AT8 immunonegative neurons in PD groups. Data: mean ± standard deviation. AFU = arbitrary fluorescence units.

We know from our previous studies that experimental and human PD are associated with loss of dopaminergic phenotype in nigrostriatal neurons and believed, at the time, that this down-regulation was associated with α-syn.^19–21^ Based upon the data described here, we now hypothesize that this down-regulation can also be tau dependent. To test this hypothesis, co-localization and quantitative analysis of TH immunoreactivity was performed in nigral neurons with or without tau aggregates from NMD, MMD, MMD-LB and PD groups. Double labelling revealed that the staining intensity of perikaryal TH immunoreactivity in nigral neurons without tau aggregates was similar across all four groups (Fig. 6). In contrast, nigral neurons with tau aggregates displayed reductions of TH immunoreactivity in subjects with MMD (Fig. 6D–F), MMD-LB (Fig. 6G–I) and PD (Fig. 6J–L). Quantitatively, an ANOVA revealed a significant difference in the optical density of TH immunoreactivity in nigral perikarya (*P* < 0.0001) across groups. The fluorescence intensity of TH staining was reduced by 15.25–21.52% in neurons without tau aggregates in the MMD, MMD-LB and PD groups, and this was not statistically different from the NMD group (*P* > 0.05). Interestingly in the MMD, MMD-LB and PD groups, nigral neurons that contained tau aggregates showed a significant reduction in TH immunofluorescence intensity (70.83% for MMD, 69.61% for MMD-LB and 70.88% for PD) relative to the NMD group (*P* < 0.001; Fig. 6M).

**Figure 7:**
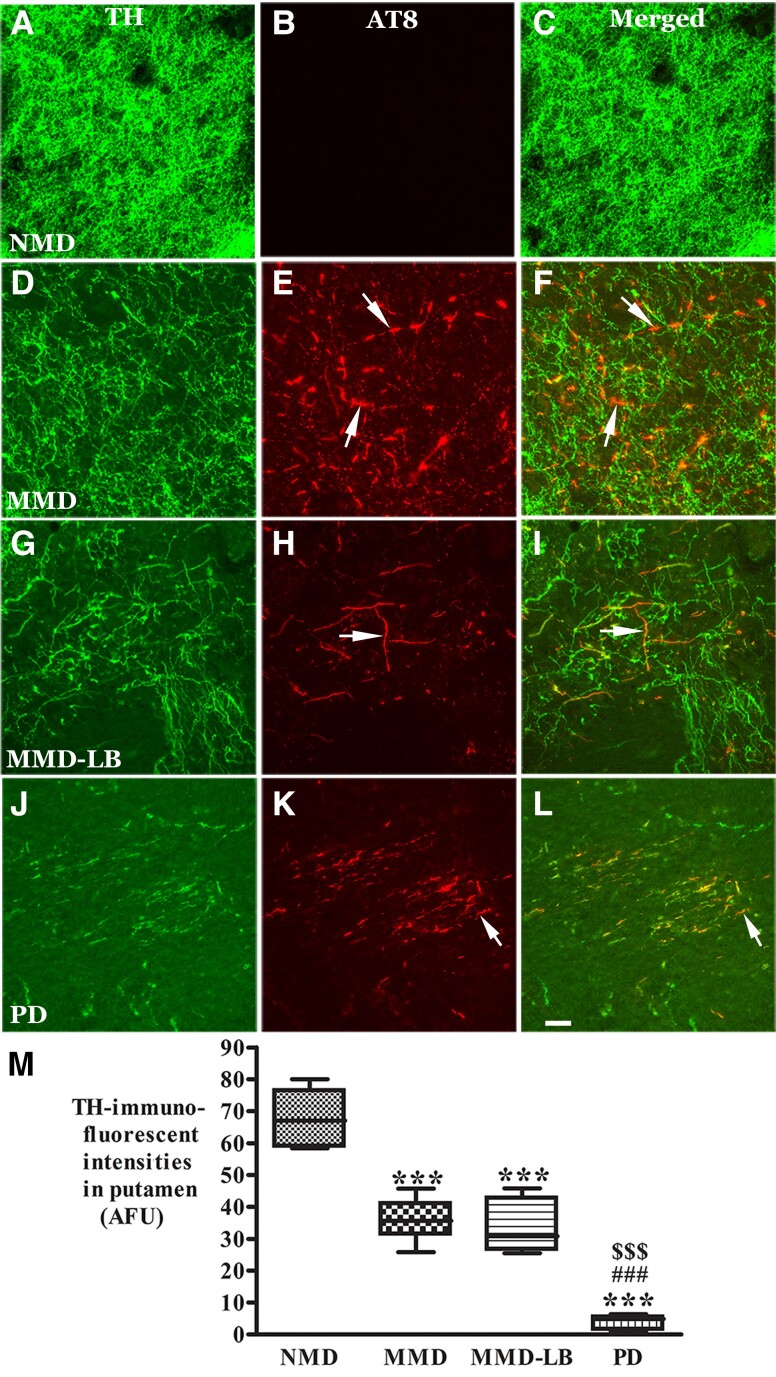
**Reduction of tyrosine hydroxylase levels in putamen with AT8-immunorepressive aggregates.** Confocal microscopic images of putamen from no motor deficit (NMD; **A**–**C**), minimal motor deficits (MMD; **D**–**F**), minimal motor deficits with nigral Lewy body pathology (MMD-LB; **G**–**I**) and Parkinson’s disease (PD; **J**–**L**) illustrated immunostaining for tyrosine hydroxylase (TH; green; **A**, **D**, **G** and **J**), AT8 (red; **B**, **E**, **H** and **K**) and co-localization of TH and AT8 (merged; **C**, **F**, **I** and **L**). Note that TH immunofluorescent intensity was extensively reduced in MMD (**D**), MMD-LB (**G**) and PD groups (**L**) relative to NMD group (**A**). The AT8-labelled threads and punctuated dots (arrows; **E**, **H** and **K**) were TH immunonegative (arrows; **F**, **I** and **L**). Scale bar in **L** = 40 µm (applies to all panels). Measurements of TH-immunorepressive (ir) intensity (**M**) further revealed that TH-ir fibres were significantly reduced in the putamen with tau aggregates in MMD, MMD-LB and PD group. ***P < 0.001 related to NMD group, ^###^*P* < 0.001 related to MMD group and ^$$$^*P* < 0.001 related to MMD-LB group. Data: mean ± standard deviation. AFU = arbitrary fluorescence units.

Within the putamen, a higher density of TH-labelled dopaminergic axons and terminals and no tau labelling were observed in NMD subjects (Fig. 7A–C). The TH-labelling was variable across the putamen in MMD (Fig. 7D) and MMD-LB (Fig. 7G). Some areas displayed TH-ir fibres with diminished intensity, while others exhibited undetectable TH immunoreactivity compared with NMD. PD (Fig. 7J) presented severe reductions of TH immunofluorescent intensity. TH immunoreactivity was not detected in AT8-labled threads and puncta in all subjects with tau pathology (Fig. 7D–L). Quantitative analyses revealed that the percentage of TH-labelled profiles within the putamen was reduced 44.41% in MMD, 49.26% in MMD-LB and 75.56% in PD relative to NMD (Fig. 7M). An ANOVA revealed a statistically significant difference across these groups with respect to decreases in TH intensity (*P* < 0.0001). *Post hoc* analyses demonstrated that the intensity of TH-labelled fibres in the putamen was significantly reduced in MMD (*P* < 0.001), MMD-LB (*P* < 0.001) and PD (*P* < 0.001) compared with the NMD group, between MMD and PD (*P* < 0.001) and between MMD-LB and PD (*P* < 0.001). In contrast, there was no significant difference between the MMD and MMD-LB groups (*P* > 0.05). These data again indicate that tau, in addition to α-syn, drives the downregulation of TH in nigral neurons.

## Discussion

Lewy pathology and dopaminergic neuronal loss in the substantia nigra are defining pathologies in PD.^3,36^ However, several reports indicated that significant neurodegeneration and cellular dysfunction precede Lewy pathology emerging in the substantia nigra, suggesting these two events may not be pathologically linked.^3,4,6,37^ Furthermore, there are emerging data from PD patients with mutations in LRRK2^5,38^ and other cases^8^ that support clinical PD without α-syn pathology. In further support of this concept, subjects with Braak PD Stages 1 and 2 exhibit dopaminergic neuronal dysfunction and neuronal loss, even though Lewy body pathology is undetectable in the substantia nigra.^3,4^

In the present study, we selected subjects with minimal parkinsonian signs, who were well characterized clinically as having parkinsonism but not PD. In all of our cases, a board-certified neuropathologist excluded other neurodegenerative diseases as contributory to our findings, although we cannot conclusively rule out an ageing frailty phenotype. In contrast to our previous studies, in which the cases were preselected based on the presence of both clinical signs and Lewy pathology, this study was primarily based upon the presence of clinical signs, with cases then further segregated according to the presence or absence of Lewy pathology.

Seventeen cases with nigral Lewy pathology were pathologically classified as MMD-LB.^39^ Eleven cases had parkinsonism, but without nigral Lewy pathology, and were classified as MMD. It should be noted that some of these cases had Lewy pathology in limbic and cortical areas. Both MMD and MMD-LB groups with minimal clinical parkinsonian signs displayed intermediate reductions of TH-ir nigral neuron density and putamenal fibres relative to NMD controls and PD subjects. Critically, there was no statistically significant difference between subjects with clinical MMD regardless of whether they had Lewy pathology or not, indicating that clinical parkinsonian syndrome and pathology can occur independent of Lewy pathology. Similarly, we investigated whether Lewy pathology was responsible for the downregulation of TH expression in substantia nigra perikarya and putamenal fibres during the development of PD. The present study also revealed that all brains with MMD had a decrease in their dopaminergic phenotype, and these decreases in TH expression were identical in cases with and without nigral Lewy pathology, indicating further that Lewy pathology is not required to produce this form of degeneration.

What is the cellular insult causing nigral dopaminergic neuronal dysfunction and death that may antedate Lewy body formation and maturation? Several pathologies including tau, α-syn, β-amyloid plaques, TDP-43, microinfarcts, atherosclerosis and cerebral amyloid angiopathy have been associated with progression of parkinsonism in ageing.^6^ Tau and α-syn are abundant brain proteins with distinct intraneuronal distribution and biological functions. Tau, a microtubule binding protein, is localized to axons, where it stabilizes and promotes microtubule polymerization, whereas α-syn is mainly found in axon terminals where it may regulate synaptic functions. If these proteins are important, their loss to pathology must be consequential. Interesting reports from Alzheimer’s disease studies demonstrated that α-syn pathology was found in Alzheimer’s disease brains.^40,41^

### Does abnormal tau modulate α-syn pathology?

We hypothesized that phosphorylated tau accumulation appears prior to a-syn pathology and may initiate nigral dopaminergic neurodegeneration, as previous studies have demonstrated neurofibrillary tangles in the substantia nigra of the elderly.^12^ We observed pathological phospho-tau in all cases with MMD regardless of whether they had Lewy pathology. In fact, the degree of tauopathy was similar in MMD and MMD-LB subjects, suggesting that tau pathology, and not α-syn pathology, may be the common denominator for what initiates the robust degeneration of the nigrostriatal pathway from the premotor stage to PD. This is supported by the relatively lower densities of nigral tau-ir aggregates and putamenal tau-ir dots and threads in the PD group relative to the MMD and MMD-LB groups. At later stages of PD (Hoehn and Yahr Stage 5), tau-ir aggregates were rarely detected in the nigrostriatal system. These nigrostriatal tau-ir aggregates in later stages of PD may disappear concomitant with dopaminergic neuronal death. Tau aggregates accumulated in the nigrostriatal system in parkinsonism without nigral Lewy body pathology, suggesting that tau accumulation may be upstream of α-syn aggregates. Although the tau aggregates are not considered a prominent feature in PD pathogenesis.^42^ the prominent expression in this parkinsonism population warrants a re-examination of the sequencing of pathogenic events from preclinical, prodromal to manifest PD. Several studies reported that tau alone was sufficient to provoke severe neurodegeneration leading to parkinsonism in the absence of synucleinopathy in frontotemporal dementia and postencephalitic parkinsonism subjects.^43–45^ Together, these data support a mechanism where phosphorylated-tau accumulations may be an important, and indeed early, pathogenic contributor to PD, although the requirement for tau leading to a nigral synucleinopathy will need to be established.^31^

What are the features of the tauopathy seen in these subjects with mild motor deficits? There are multiple isoforms of tau, with 3R-tau and 4R-tau being most prominent in humans. Both tau isoforms were seen in these cases and co-localized with phosphorylated tau marker AT8, indicating that the expression of 4R-tau was increased and subsequently phosphorylated. In the healthy brain, the 3R and 4R isoforms are equivalently expressed.^46^ In Alzheimer’s brain, there is a decrease in 3R tau isoform or increase in 4R tau levels resulting in shift in the ratio of 4R-tau to 3R-tau.^47,48^ Our observation demonstrated that light 3R and intense 4R labelled neurons observed in substantia nigra of MMD and PD were similar to a pattern that existed in temporal cortex of Alzheimer’s disease (Supplementary Fig. 6), indicating that unequal 3R and 4R tau expression was associated with neurodegeneration.

Lower total tau levels in CSF have been associated with increased motor severity in early-stage PD.^49^ AT8 labelled tau may be related to insoluble tau in this study. We did not have the appropriate frozen material to test this hypothesis, but whether tau accumulation and aggregation in nigral dopaminergic neurons are associated with tau isoform imbalances or reduction of soluble tau deserves further study. Tau and α-syn are both intrinsically unfolded proteins that can adopt different conformations including oligomers and abnormal intracellular aggregates under pathological conditions. The disorders with tauopathies are commonly accompanied with parkinsonian signs,^50^ while disorders with synucleinopathies are commonly attended by dementia.^24^ Tau aggregates have been described in familial PD linked to A53T α-syn mutation^51–53^ and in LRRK2 G2019S variant carriers.^38^ Our observations demonstrate that all MMD-LB cases have both pathologies as well as, in our cohort, the vast majority of sporadic PD cases. Thus, while our MMD cases suggest that tau is critical for early manifestation of the disease, we cannot currently rule out an interaction in later disease. This morphological feature demonstrated that tau and α-syn may interact and the interaction may play an important role on the development and spreading of neurodegeneration.^54,55^

Mixed brain pathologies accelerate progression of PD development.^6^ Cellular models have verified that pre-formed α-syn fibrils cross-seed intracellular tau to induce neurofibrillary tangle formation.^56^ Alpha-syn phosphorylation triggers tau pathogenicity and induces widespread phosphorylated tau with prion-like nature in various brain areas.^54^ High levels of S396 phospho-tau and phospho-α-syn were found in synaptic-enriched fractions of the frontal cortex in Alzheimer’s disease and PD.^57^ The SNCA gene polymorphism rs2572324 has been reported to be associated with both neocortical Lewy pathology and neurofibrillary tangles.^58^ Our results support the thesis that α-syn and tau form a deleterious feed-forward process essential for the development and spreading of neurodegeneration in PD, and this occurs in a subset of subjects with parkinsonism.

GWAS revealed that single nucleotide polymorphisms within tau and α-syn have the highest association with PD.^17,18,59^ Our results demonstrated that most tau aggregates do not co-localize with α-syn aggregates and, in fact, a subpopulation of MMD subjects do not have nigral α-syn at all. Even if α-syn and tau aggregates occurred in the same brain, the formed aggregates were for the most part spatially separated. These data suggest there was no direct relationship between tau and α-syn aggregates in morphological analyses.

The misfolded protein accumulation and aggregation may be initially associated with other factors such as inefficiency of axonal transport. Tau is synthesized in nigral perikarya and transported to the distal portions of axons, where it provides microtubule stabilization and flexibility as needed.^60^ Tau is normally undetectable in perikarya and dendrites.^61^ Our morphological analyses revealed phosphorylated tau accumulations ranging from a few soluble granules to filling the nigral perikarya and processes with insoluble protein. Supported by these findings, we speculate that tau accumulation within perikarya, dendrites and axons is associated with dysfunction of axonal transport. In our previous studies of humans and PD models, we demonstrated decreases in the anterograde and retrograde axoplasmic transport motors, kinesin light chain and dynein, respectively, are associated with α-syn aggregation.^31^ Whether phosphorylated and aggregated tau are associated with dysfunction of axonal transport warrants further study.

In summary, the present series of observations is most consistent with pathological tau being part of an early, pre-synuclein, process of nigrostriatal degeneration in premotor PD. Critically, this proposed mechanism hinges on MMD cases being truly a precursor to PD. The clinical and pathological data support this view; MMD cases are intermediate between NMD and PD in motor impairment, TH-ir nigral cell loss and loss of TH-ir putamenal innervation, as well as nigral and putamenal phenotypic TH down-regulation. All these pathological events are associated with tau pathology but not always α-syn, as they occur equally in MMD cases with and without Lewy pathology. These data should change our thinking of PD pathology and therapies directed towards reducing pathological tau or increasing soluble tau might be needed either alone or in combination with α-syn therapies. Furthermore, the use of an MMD cohort which displays robust clinical changes and pathological alteration on tau PET or phospho-tau and total tau levels in CSF might be a cohort that would benefit most from such a clinical trial.

## Supplementary Material

awad388_Supplementary_Data

## Data Availability

The data that supports the findings of this study is available at doi.org/10.5281/zenodo.10268773.
