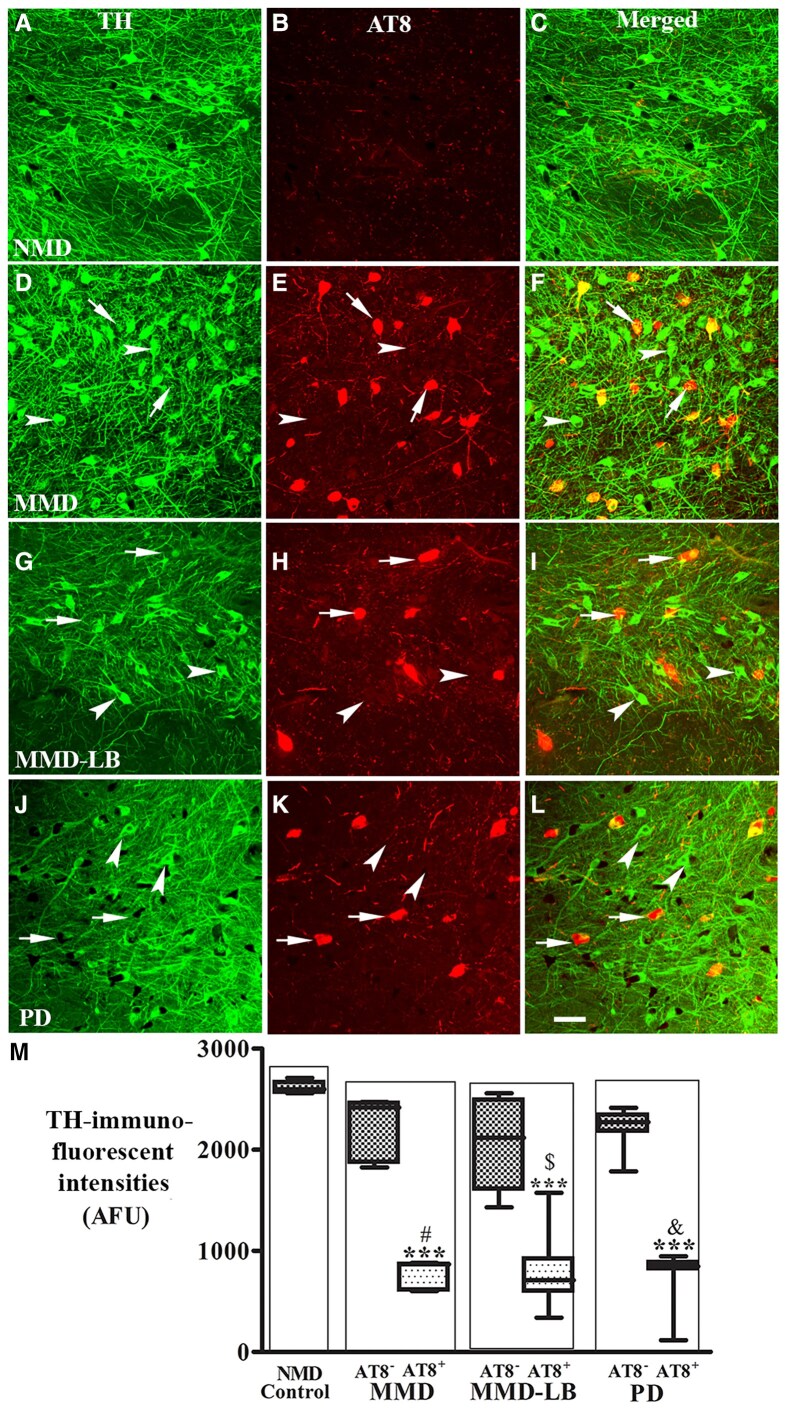# Correction to: Nigrostriatal tau pathology in parkinsonism and Parkinson's disease

**DOI:** 10.1093/brain/awaf146

**Published:** 2025-04-29

Yaping Chu, Warren D. Hirst, Howard J. Federoff, Ashley S. Harms, A. Jon Stoessl, Jeffrey H. Kordower, Nigrostriatal tau pathology in parkinsonism and Parkinson's disease, *Brain*. 2024;147:444-457. https://doi.org/10.1093/brain/awad388

The authors apologize for errors in Fig. 6G–I, where panels G–I were a duplicate of Fig. 6D–F. In panel M, the *y*-axis label has been corrected from ‘TH-immunofluoresecent intensities (AFU)’ to ‘TH-immunofluorescent intensities (AFU)’.

The corrected figure is provided only in this correction notice to preserve the published version of record. These corrections do not change the manuscript's description, interpretation or original conclusions.

Corrected Figure 6:

**Figure:**