# Supplementary material for: Nigrostriatal tau pathology in parkinsonism and Parkinson’s disease
Source: Brain. 2023 Nov 25;147(2):444–57. doi: 10.1093/brain/awad388 (PMC10834249; doi:10.1093/brain/awad388)
Supplement: awad388_Supplementary_Data [file awad388_supplementary_data.pdf]

Evaluating densities of TH immunoreactive neurons and AT8 and p-S129 immunoreactive aggregates in substantia nigra

The density of nigral TH-immunoreactive (TH-ir) neurons and misfolded proteins were estimated for each subject using stereology[1]. All stereological estimates were separately counted using a uniform, systematic, and random design. An optical fractionator unbiased sampling design was used and a Cavalieri's principle to assess the volume within substantia nigra and putamen.[2–4] In each subject, we evaluated the substantia nigra pars compacta from the level of the midbrain at the exit of the 3rd nerve to the decussation of the superior cerebellar peduncle. Approximately 5 equispaced sections were sampled from each brain. The section sampling fraction (ssf) was 1/0.055. The distance between sections was approximately 0.72 mm. In cross-section, the substantia nigra is in the ventral midbrain. The substantia nigra pars compacta was outlined using a 1.25× objective. A systematic sample of the area occupied by the substantia nigra pars compacta was made from a random starting point (StereoInvestigator software v2021.1.3; RRID:SCR_018948; https://www.mbfbioscience.com/stereo-investigator). Counts were made at regular predetermined intervals (x = 313µm, y = 313µm), and a counting frame (70 × 70µm = 4900 µm^2) was superimposed on images obtained from tissue sections. The area sampling fraction (asf) was 1/0.05. These sections were then analyzed using a 60× Planapo oil immersion objective with a 1.4 numerical aperture. The section's thickness was empirically determined. Briefly, as the top of the section was first brought into focus, the stage was zeroed at the z-axis by software. The stage then stepped through the z-axis until the bottom of the section was in focus. Section thickness averaged 16.21 ± 2.3µm in the midbrain. The dissector height (counting frame thickness) was 10 µm. This method allowed for 1µm top guard zones and at least 2µm bottom guard zones. The thickness sampling fraction (tsf) was 1/0.62. Care was taken to ensure that the top and bottom

forbidden planes were never included in the cell counting. The ultimate estimate of the counted profiles within the substantia nigra pars compacta was calculated separately using the following formula: $N=\Sigma Q^{-} \cdot 1/ssf \cdot 1/asf \cdot 1/tsf$. ΣQ^{-} was the estimated number of raw counts.

The Cavalieri estimator (StereoInvestigator software v2021.1.3; RRID:SCR_018948; https://www.mbfbioscience.com/stereo-investigator) was used to estimate volume of the substantia nigra pars compacta on immunostained sections. We sampled serial sections of substantia nigra that extended from the exit of the 3rd nerve to the decussation of the superior cerebellar peduncle using the optical fractionator principle described above. The distance between sections interspace was approximately 0.72 mm. According to NM the substantia nigra is outlined in cross-section using a 1.25x objective. The section thickness was empirically determined. Section thickness averaged 16.23±2.2μm. The area estimation of substantia nigra pars compacta was performed by means of a 50 x 50 μm point grid with 10x objective. The total volume of substantia nigra pars compacta was calculated by Cavalieri estimator software.[4,5] The coefficients of error (CE) were calculated according to the procedure of Gunderson and colleagues as estimates of precision.[6,7] The values of CE were 0.12 ± 0.05 (range 0.10 to 0.15) in PD and 0.10 ± 0.02 (range 0.08 to 0.12) in MMD and NMD. The AT8-ir and p-S129-ir threats and particles in putamen were evaluated using the above-described methods.

Evaluating TH immunoreactive area and fiber thickness in putamen

Measurements for TH-ir area were performed with particle analyses program and for TH-ir fiber thickness were performed using Feret's Diameter of ImageJ (v1.47, RRID:SCR_003070, https://imagej.net/).[8] Using stereological principles, the putamen was outlined with 1.25x objective on 5 stained sections through rostral to caudal putamen and more than 25 images were randomly

sampled with 20x objective for each subject. The black and white images taken from immunostained putamenal sections and were stored in tif file. The set scale was 2.254 pixels/micron. All images were threshold at default and analyzed particles at 1 pixel size or above. The area of TH-ir products were calibrated in square millimeter (mm^2) and the thickness of TH-ir fibers were calibrated in micrometer.

Double and triple staining and evaluating optical densities of immunofluorescence.

A double-label immunofluorescence procedure was employed to determine whether tau aggregates co-existed in neurons that expressed α-syn and whether tau aggregates affected the dopaminergic neuronal functions[9]. Midbrain and putamenal sections were incubated in the first primary antibody AT8 overnight and the goat anti-mouse antibody coupled to DyLight 649 (1:200, Jackson ImmunoResearch, Cat# 115-495-146, RRID:AB_3076197) for 1 h. After blockade for 1 h, the sections were then incubated in the second primary antibodies (p-S129, Abcam, Cat# ab51253, RRID:AB_869973, 1:500; α-syn, Abcam, Cat# ab138501, RRID:AB_2537217, 1:500; TH, Pel-Freez Biologicals, Cat# P40101, RRID:AB_2313713, 1:1000) overnight, and the goat anti-rabbit antibody coupled to DyLight 488 (1:200, Vector Laboratories, Cat# DI-1488, RRID:AB_2336402) for 1 h. The stained sections were mounted on gelatin-coated slides, dehydrated through graded alcohol, cleared in xylene, and covered using DPX (Sigma-Aldrich). A triple-label immunofluorescence procedure was employed to examine three and four repeat (3R, 4R) tau isoforms and their colocalization with AT8. Midbrain sections were incubated overnight in primary antibodies: 3R-tau (rat monoclonal, 1:500, FUJIFILM Wako Shibayagi, Cat# 016-26581, RRID:AB_3076196), 4R-tau (rabbit monoclonal, 1:500, Abcam, Cat# ab218314, RRID:AB_3068614), and AT8 (mouse monoclonal, 1:1000,Thermo Fisher Scientific, Cat#

MN1020, RRID:AB_223647) and for 2 hours in secondary antibodies: Cy2 conjugated goat-anti rat (1:200, Jackson ImmunoResearch Labs, Cat# 112-225-167, RRID:AB_2338278), Cy3 conjugated goat-anti rabbit (1:200, Jackson ImmunoResearch Labs, Cat# 115-175-146, RRID:AB_2338713), and Cy5 conjugated goat-anti mouse (1:200, Jackson ImmunoResearch Labs, Cat# 111-165-003, RRID:AB_2338000). The stained sections were mounted on gelatin-coated slides, dehydrated through graded alcohol, cleared in xylene, and covered using DPX (Sigma-Aldrich, Cat# 06522).

Fluorescence intensity measurements were performed according to our previously published procedures. [3,10] All immunofluorescence labeled images were scanned with an Olympus Confocal Fluoview microscope equipped with argon, helium-neon lasers, and transparent optics. At 20× magnification objective and a 488, 543, and 633 nm excitation source, images were acquired at each sampling site in the substantia nigra pars compacta and were saved to a Fluoview file (Olympus Fluoview FV10-ASW v4.2b, RRID:SCR_014215, https://www.olympus-lifescience.com/). Following acquisition of an image, the stage moves to the next sampling site to ensure a completely non-redundant evaluation. Once all images were acquired, optical density measurements were performed on individual nigral neurons. To maintain consistency of the scanned image for each slide, the laser intensity, confocal aperture, photomultiplier voltage, offset, electronic gain, scan speed, image size, filter and zoom were set for the background level whereby autofluorescence was not visible with a control section. These settings were maintained throughout the entire experiment.[3] The intensity mapping sliders ranged from 0 to 4095; 0 represented a maximum black image and 4095 represented a maximum bright image. The TH-ir perikarya with or without AT8-ir aggregates were identified and outlined separately. Quantitative optical density of immunofluorescence was performed on individual TH-ir soma with or without AT8-ir

aggregates in different channels. Five equispaced sections of the substantia nigra were sampled and evaluated. The number of cells per case was analyzed as follows: 50–70 nigral cells that contained AT8-ir aggregates and >100 nigral cells that did not contain aggregates per subject. To account for differences in background staining intensity, five background intensity measurements lacking immunofluorescent profiles were taken from each section. The mean of these five measurements constituted the background intensity that was then subtracted from the measured optical density of each individual neuron to provide a final optical density value. To confirm co-localization of the AT8 and p-s129-α-syn immunofluorescence, optical scanning through the neuron's z-axis was performed at 1-μm thickness and neurons suspected of being double labeled were confirmed with confocal cross-sectional analyses. The optical density of TH-immunofluorescent intensities in putamen was determined using Image J program described above.

References

1. Mehrabi NF, Singh-Bains MK, Waldvogel HJ, Faull RLM. Stereological Methods to Quantify Cell Loss in the Huntington's Disease Human Brain. In: ; 2018:1-16. doi:10.1007/978-1-4939-7825-0_1
2. Chu Y, Kompoliti K, Cochran EJ, Mufson EJ, Kordower JH. Age-related decreases in Nurr1 immunoreactivity in the human substantia nigra. *J Comp Neurol*. 2002;450(3):203-214. doi:10.1002/cne.10261
3. Chu Y, Morfini GA, Langhamer LB, He Y, Brady ST, Kordower JH. Alterations in axonal transport motor proteins in sporadic and experimental Parkinson's disease. *Brain*. 2012;135(Pt 7):2058-2073. doi:10.1093/brain/aws133
4. Gundersen HJG, Jensen EB. The efficiency of systematic sampling in stereology and its prediction*. *J Microsc*. 1987;147(3):229-263. doi:10.1111/j.1365-2818.1987.tb02837.x
5. Chu Y, Le W, Kompoliti K, Jankovic J, Mufson EJ, Kordower JH. Nurr1 in Parkinson's disease and related disorders. *J Comp Neurol*. 2006;494(3):495-514. doi:10.1002/cne.20828
6. Schmitz C, Hof PR. Recommendations for straightforward and rigorous methods of counting neurons based on a computer simulation approach. *J Chem Neuroanat*. 2000;20(1):93-114. doi:10.1016/S0891-0618(00)00066-1
7. West MJ, Gundersen HJG. Unbiased stereological estimation of the number of neurons in the human hippocampus. *J Comp Neurol*. 1990;296(1):1-22. doi:10.1002/cne.902960102

8. Igathinathane C, Pordesimo LO, Columbus EP, Batchelor WD, Methuku SR. Shape identification and particles size distribution from basic shape parameters using ImageJ. *Comput Electron Agric*. 2008;63(2):168-182. doi:10.1016/j.compag.2008.02.007
9. Kordower JH, Chu Y. Immunofluorescence Multi-label Protocol for Free-floating Fixed Tissue. *protocols.io*. Published online 2023. doi:10.17504/protocols.io.j8nlkorn5v5r/v1
10. Chu Y, Goldman JG, Kelly L, He Y, Waliczek T, Kordower JH. Abnormal alpha-synuclein reduces nigral voltage-dependent anion channel 1 in sporadic and experimental Parkinson's disease. *Neurobiol Dis*. 2014;69:1-14. doi:10.1016/j.nbd.2014.05.003

Supplementary Table 1. Demographics of older adults with no motor deficit (NMD)

Case no.	Age (years)	Gender	PMI (hour)	Glob-P (0-100)	Gait (0-100)	Rigidity (0-100)	Tremor (0-100)	Bradykinesia (0-100)
B00-76	71	M	4.08	6.51	0	26.06	0	0
B97-32	73	M	7.25	3.47	0	0	13.91	0
B98-115	84	M	4.00	2.40	9.63	0	0	0
B96-98	72	M	7.00	3.03	12.14	0	0	0
B98-84	78	F	3.00	2.58	0	10.35	0	0
B00-59	91	F	10.66	4.90	0	0	0	19.66
B99-07	84	F	3.50	2.14	8.57	0	0	0
B96-50	88	F	7.00	6.35	25.42	0	0	0
AT7-1	91	F	6.50	7.58	30.32	0	0	0
**Mean±SD**	**81.33±8.06**	**4/5**	**5.88±2.45**	**4.32±2.05**	**9.56±11.48**	**4.04±8.93**	**1.54±4.63**	**2.18±6.55**

PMI, postmortem interval; MMSE, Glo-P, global parkinsonism.

Supplementary Table 2. Demographics of older adults with motor deficit but absent nigral Lewy body (MMD)

Case no.	Age (years)	Gender	PMI (hour)	Glob-P (0-100)	Gait (0-100)	Rigidity (0-100)	Tremor (0-100)	Bradykinesia (0-100)
AT2-5	95	F	6.17	15.80	35.71	0.00	0.00	27.50
AT3-1	97	F	10.08	21.31	66.67	0.00	6.06	12.50
AT4-1	87	F	4.92	12.5	25.00	25.00	0.00	12.50
AT5-1	96	F	12.61	32.41	82.14	20.00	0.00	27.50
AT5-3	92	M	14.17	29.23	64.29	10.00	15.15	27.50
AT5-4	92	F	7.41	9.29	32.14	0.00	0.00	5.00
AT6-1	90	F	18.75	34.82	64.29	30.00	0.00	45.00
AT6-3	95	F	7.1	44.01	78.57	60.00	0.00	37.50
AT6-4	93	M	12.75	23.84	64.29	0.00	6.06	25.00
AT6-5	96	F	6.5	41.60	71.43	35.00	0.00	60.00
AT6-6	90	M	9.75	14.28	42.11	0.00	0.00	15.00
**Mean±SD**	**93.00±3.10**	**3/8**	**10.24 ±4.07**	**25.37 ±11.92**	**56.96 ±19.68**	**16.36±19.76**	**2.47±4.85**	**26.81±16.05**

PMI, postmortem interval; Glo-P, global parkinsonism.

Supplementary Table 3. Demographics of older adults with minimal motor deficit and nigral Lewy body (MMD-LB)

Case no.	Age (years)	Gender	PMI (hour)	Glob-P (0-100)	Gait (0-100)	Rigidity (0-100)	Tremor (0-100)	Bradykinesia (0-100)
AT1-1	92	F	4.75	50.00	57.14	80.00	20.00	17.50
AT1-2	88	F	8.17	30.00	42.86	60.00	0.00	2.50
AT1-3	89	M	0.75	19.82	39.29	20.00	0.00	20.00
AT2-1	95	F	26.05	20.71	0.00	15.00	12.12	35.00
AT2-2	95	M	5.67	13.75	50.00	5.00	0.00	0.00
AT2-3	90	F	6.67	8.40	26.09	0.00	0.00	7.50
AT2-4	97	M	4.75	15.95	57.14	25.00	0.00	22.86
AT2-6	100	F	6.33	16.19	75.00	10.00	6.06	32.50
AT2-7	85	M	6.50	9.91	32.14	5.00	0.00	2.50
AT3-2	89	M	5.33	8.70	14.29	5.00	3.03	12.50
AT3-3	90	M	13.92	37.85	67.86	30.00	6.06	47.50
AT3-4	94	F	4.40	10.87	43.48	0.00	0.00	0.00
AT3-5	93	F	13.52	5.09	17.86	0.00	0.00	2.50
AT3-6	90	M	5.58	10.35	38.13	15.00	6.06	10.00
AT4-2	82	M	17.00	8.21	17.86	0.00	6.06	15.00
AT4-3	77	M	13.90	8.75	0.00	0.00	0.00	35.00
AT4-4	91	F	16.00	3.39	3.57	5.00	0.00	5.00
**Mean±SD**	**90.41±5.53**	**9/8**	**9.37±6.37**	**16.34±12.41**	**34.27±23.15**	**16.18±22.54**	**3.49±5.56**	**15.75±14.47**

PMI, postmortem interval; Glo-P, global parkinsonism.

Supplementary Table 4. Demographics of Patients with Parkinson's Disease

Case. No	Age (years)	Gender	PMI (hour)	UPDRSIII (on)	H&Y (on)	Glob-P (0-100)	Gait (0-100)	Rigidity (0-100)	Tremor (0-100)	Bra-K (0-100)
B13-45	57	M	6.30	35.0	2	25.17	25.00	15.00	10.71	50.00
B12-95	66	M	12.00	38.0	2	32.12	20.00	25.00	42.86	40.63
B10-90	77	F	3.50	22.0	2	24.93	25.00	10.00	17.86	46.88
B09-04	74	M	8.21	38.5	2	25.47	50.00	30.00	0.00	21.88
B14-01	71	M	5.30	33.5	3	27.32	37.50	20.00	14.28	37.50
B13-46	70	F	6.00	51.0	3	46.63	60.00	60.00	7.14	59.38
B12-66	87	F	6.00	31.0	3	27.14	30.00	25.00	28.57	25.00
B10-10	77	F	5.00	53.0	3	41.69	55.00	35.00	14.29	62.50
B12-52	89	M	2.30	39.0	3	31.87	40.00	50.00	0.00	37.50
B13-49	80	M	10.00	46.0	4	44.06	70.00	50.00	0.00	56.25
B13-47	86	M	6.30	53.0	4	39.37	50.00	45.00	0.00	62.50
B13-50	72	M	7.00	54.0	5	47.18	95.00	50.00	0.00	43.75
B04-42	76	M	3.30	65.0	4	47.18	70.00	50.00	0.00	68.75
B07-11	72	F	5.00	58.0	5	54.34	66.66	65.00	0.00	85.71
B08-110	95	F	3.30	49.0	5	45.11	83.33	40.00	0.00	57.14
B09-127	76	F	4.15	44.0	4	46.09	75.00	50.00	0.00	59.38
B12-110	88	F	5.00	42.0	4	38.75	55.00	50.00	0.00	50.00
B14-04	81	M	7.30	40.0	4	33.75	50.00	35.00	0.00	50.00
B14-05	85	F	4.30	37.0	3	26.67	55.00	20.00	3.57	28.13
B16-02	74	F	10.00	40.0	4	28.56	16.66	15.00	35.71	46.87
B95-129	70	M	9.00	47.0	4	41.75	79.16	20.00	0	67.85
B96-11	77	M	5.30	42.0	3	43.75	75.00	50.00	0	50.00
B10-02	86	M	4.00	65	5	62.57	83.33	75.00	6.25	85.71
B98-09	87	F	9.00	62.0	5	45.95	79.16	35.00	12.50	57.14
**Mean±SD**	**78.04±8.69**	**13/11**	**5.99±2.41**	**45.20±10.91**	**3.58±1.01**	**38.64±10.24**	**56.07±22.34**	**38.33±17.29**	**8.07±12.27**	**52.10±16.28**

PMI, postmortem interval; MMSE, mini-mental status examination; UPDRS, United Parkinson's Disease Rating Scale; Bra-k, Bradykinesia.

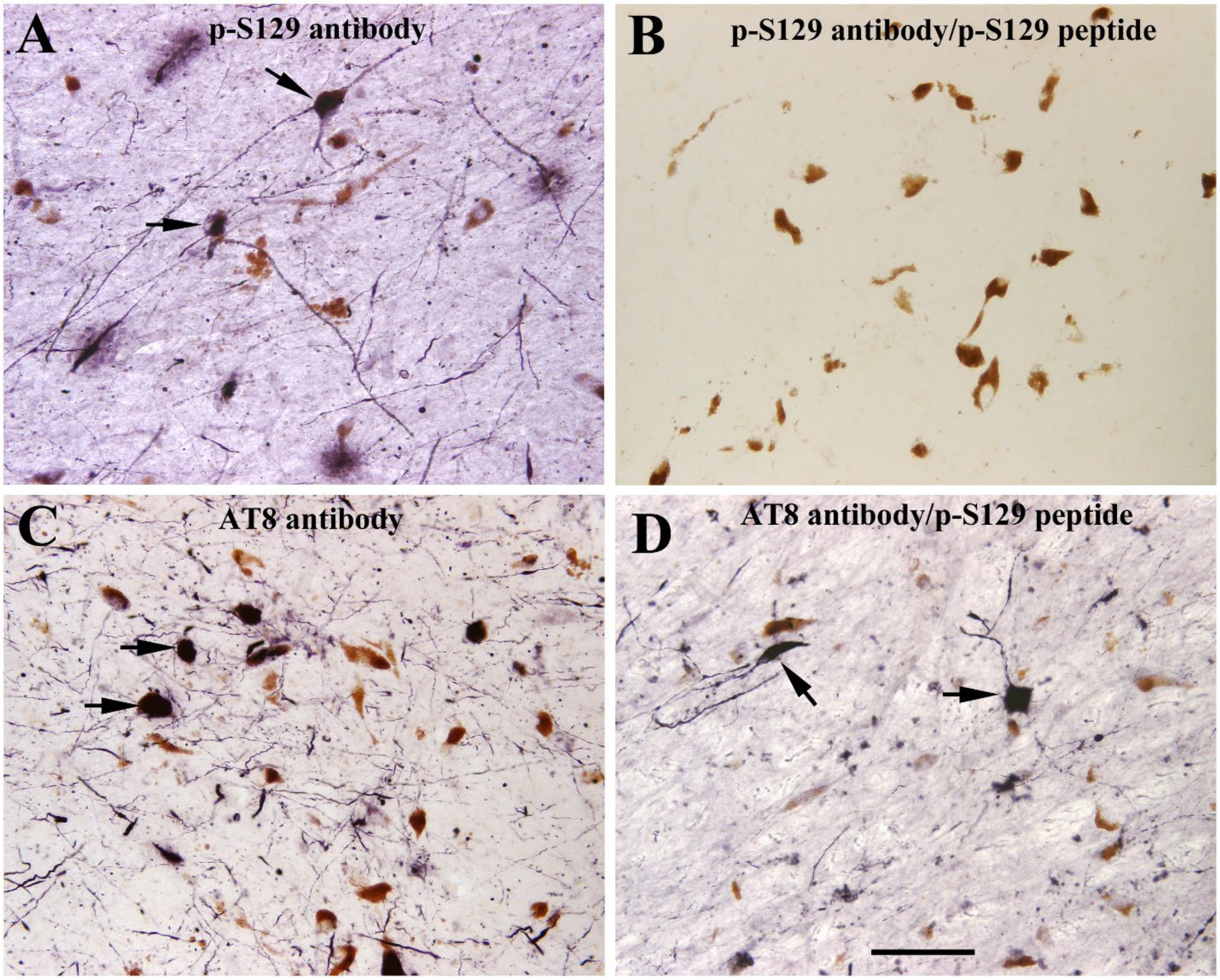

Supplementary Fig.1. Pre-adsorption of phosphor-S129 and AT8 antibodies with phospho-S129 peptide.

Photomicrographs of nigral sections from PD brain illustrated p-S129 and AT8 immunoreactivities. P-S129-immunopositive aggregates (arrows, A) were observed in nigral cells without pre-adsorption. After a pre-adsorption of p-s129 antibody/p-s129 peptide, the p-S129 staining was undetectable (B). Nigral AT8-immunopositive aggregates were observed in both tissue sections with (D) or without (C) pre-adsorption of AT8 antibody/p-S129 peptide. Scale bar in C = 100μm (applies to all).

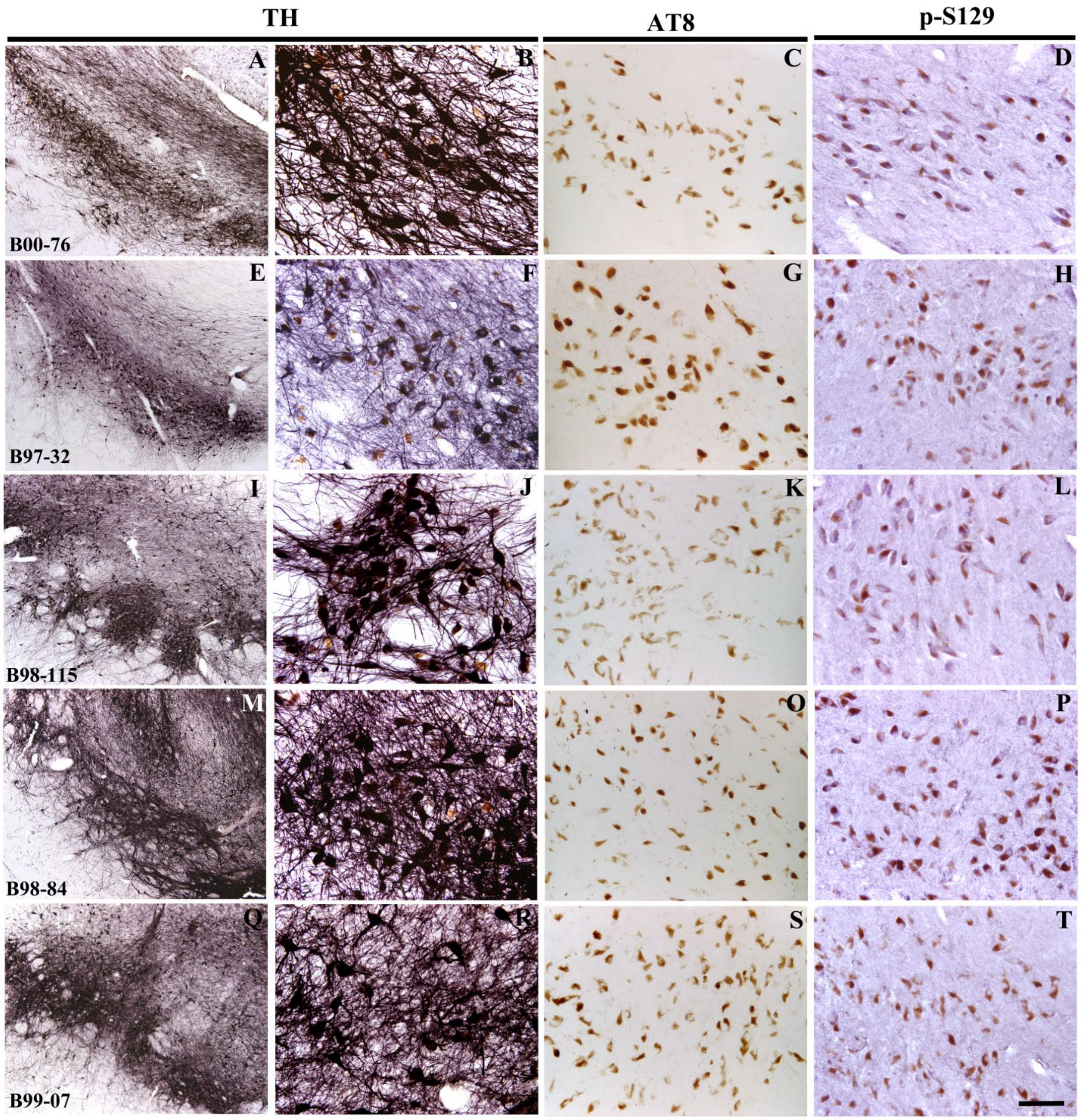

Supplementary Fig. 2. Pattern of tyrosine hydroxylase (TH), phospho-tau (AT8), and phospho-alpha-synuclein (p-S129) immunostaining in substantia nigra from subjects without motor deficits (NMD).

Photomicrographs of nigral sections illustrated TH (Left two columns), AT8 (third column), and p-S129(right column) immunostaining patterns. Note Intense and extensive TH-immunoreactive neurons with widespread local plexus of TH-immunoreactive processes. Neither AT8 nor p-S129 immunopositive inclusion was detectable. Scale bar in T =100 µm (applies to right three columns) and 500 µm for left column.

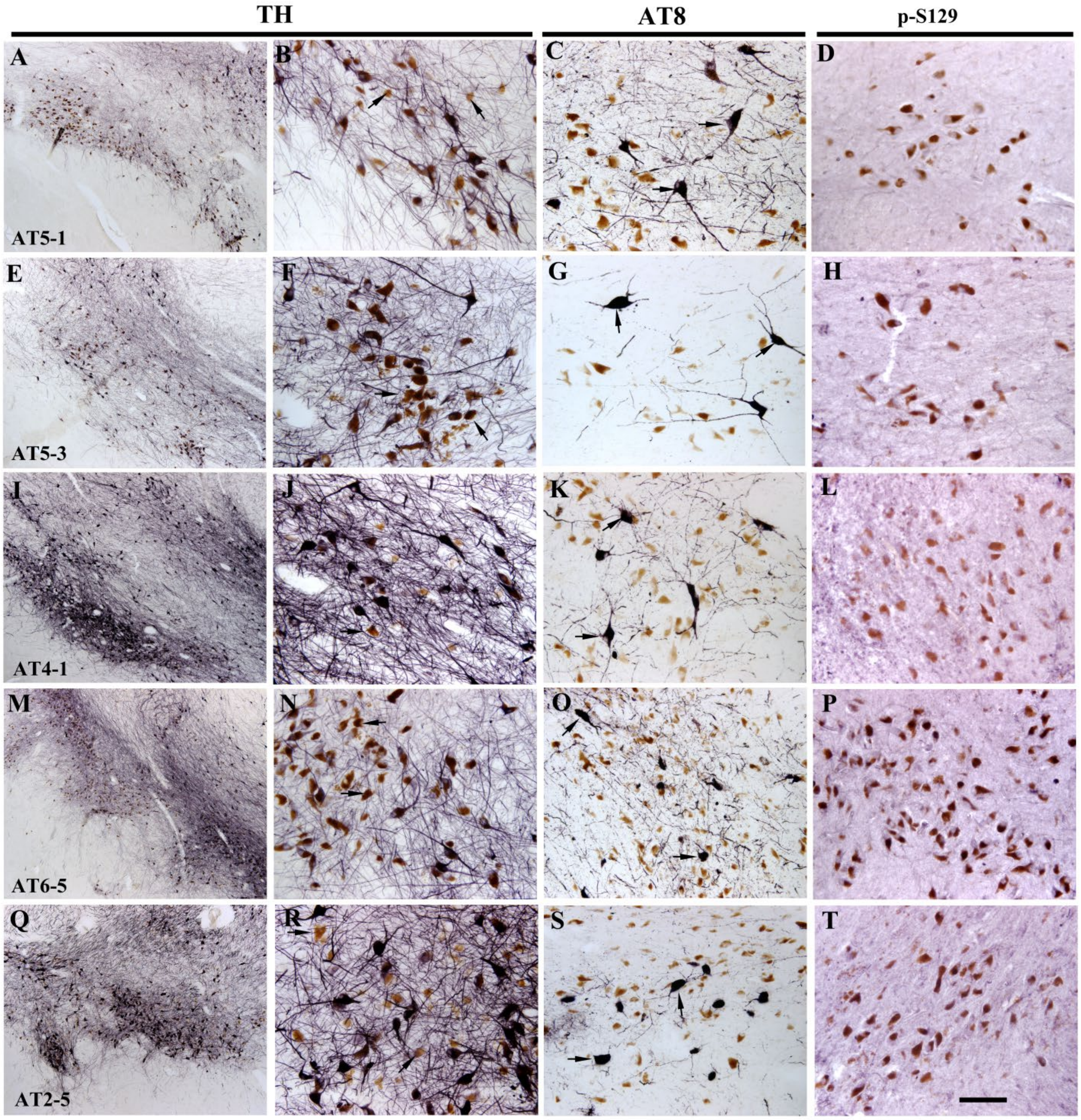

Supplementary Fig. 3. Pattern of tyrosine hydroxylase (TH), phospho-tau (AT8), and phospho-alpha-synuclein (p-S129) immunostaining in substantia nigra from subjects with motor deficits (MMD).

Photomicrographs of nigral sections illustrated TH (Left two columns), AT8 (third column), and p-S129(right column) immunostaining patterns. Note many melanized nigral neurons (brown pigmented granules) displayed TH-immunonegative (arrows, B, F, J, N, R) and AT8-immunopositive aggregates (arrows, C, G, K, O, S). Alpha-synuclein inclusion was undetectable. Scale bar in T =100 μm (applies to right three columns) and 500 μm for left column.

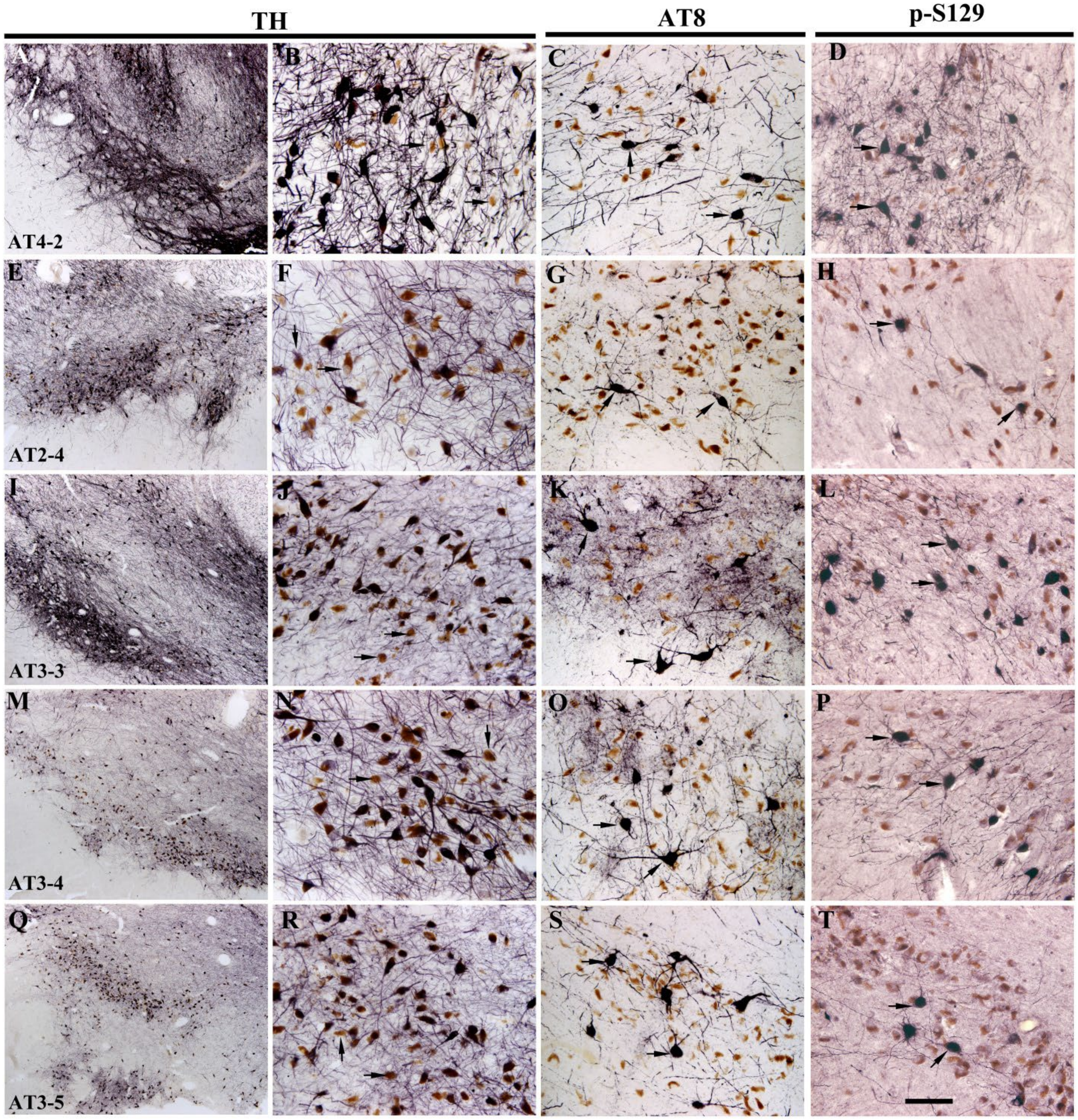

Supplementary Fig. 4. Pattern of tyrosine hydroxylase (TH), phospho-tau (AT8), and phospho-alpha-synuclein (p-S129) immunostaining in substantia nigra from subjects with motor deficits and nigral Lewy body (MMD-LB).

Photomicrographs of nigral sections illustrated TH (Left two columns), AT8 (third column), and p-S129(right column) immunostaining patterns. Note many melanized nigral neurons (brown pigmented granules) displayed TH-immunonegative (arrows, B, F, J, N, R). Both AT8- (arrows, C, G, K, O, S) and p-S129- (arrows, D, H, L, P, T) immunopositive aggregates were observed. Scale bar in T =100 μm (applies to right three columns) and 500 μm for left column.

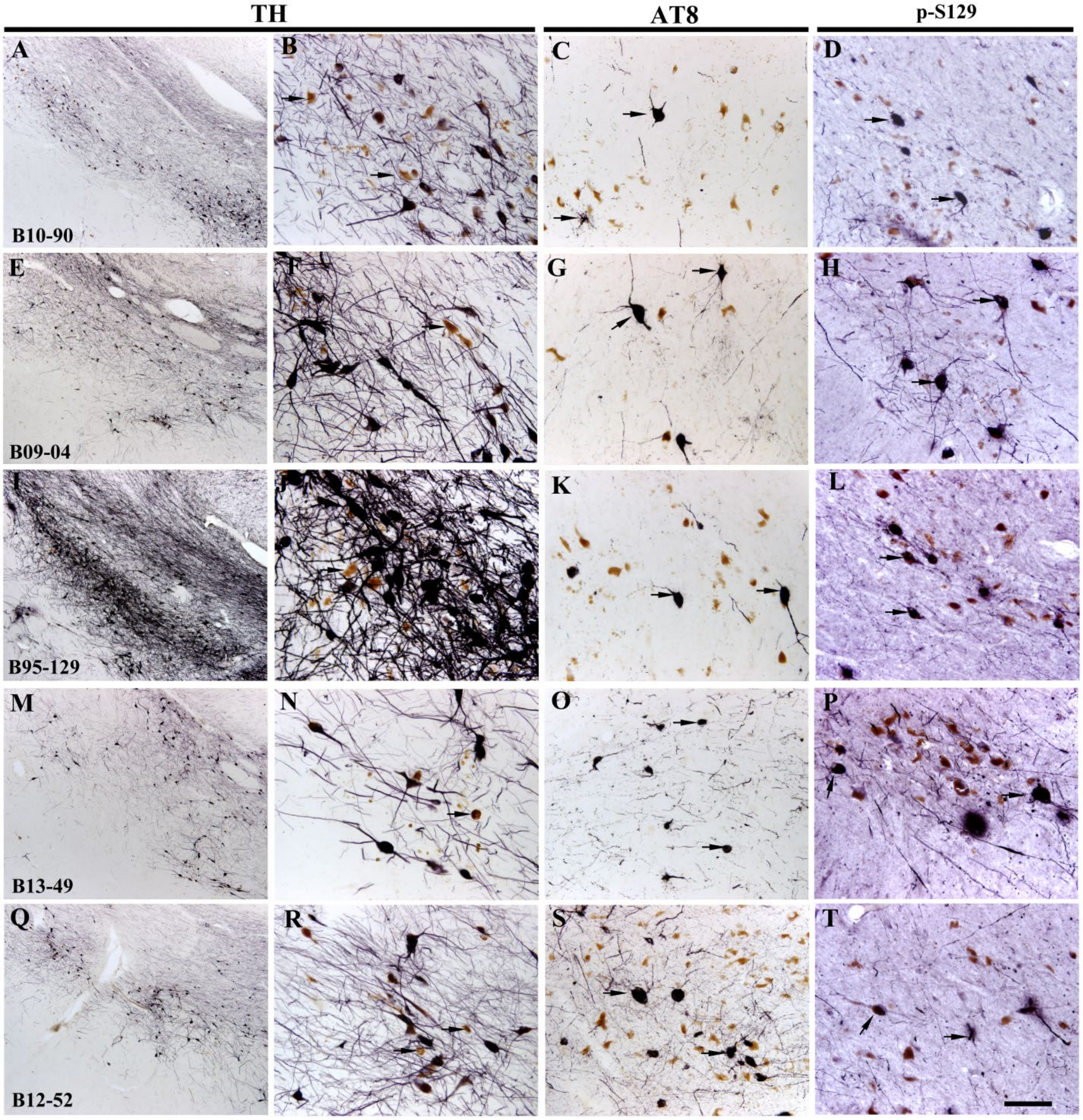

Supplementary Fig. 5. Pattern of tyrosine hydroxylase (TH), phospho-tau (AT8), and phospho-alpha-synuclein (p-s129) immunostaining in substantia nigra from subjects with Parkison's disease (PD).

Photomicrographs of nigral sections illustrated TH (Left two columns), AT8 (third column), and p-S129(right column) immunostaining patterns. Note many remaining melanized nigral neurons (brown pigmented granules) displayed TH-immunonegative (arrows, B, F, J, N, R). Both AT8- (arrows, C, G, K, O, S) and p-S129- (arrows, D, H, L, P, T) immunopositive aggregates were observed. Scale bar in T =100 μm (applies to right three columns) and 500 μm for left column.

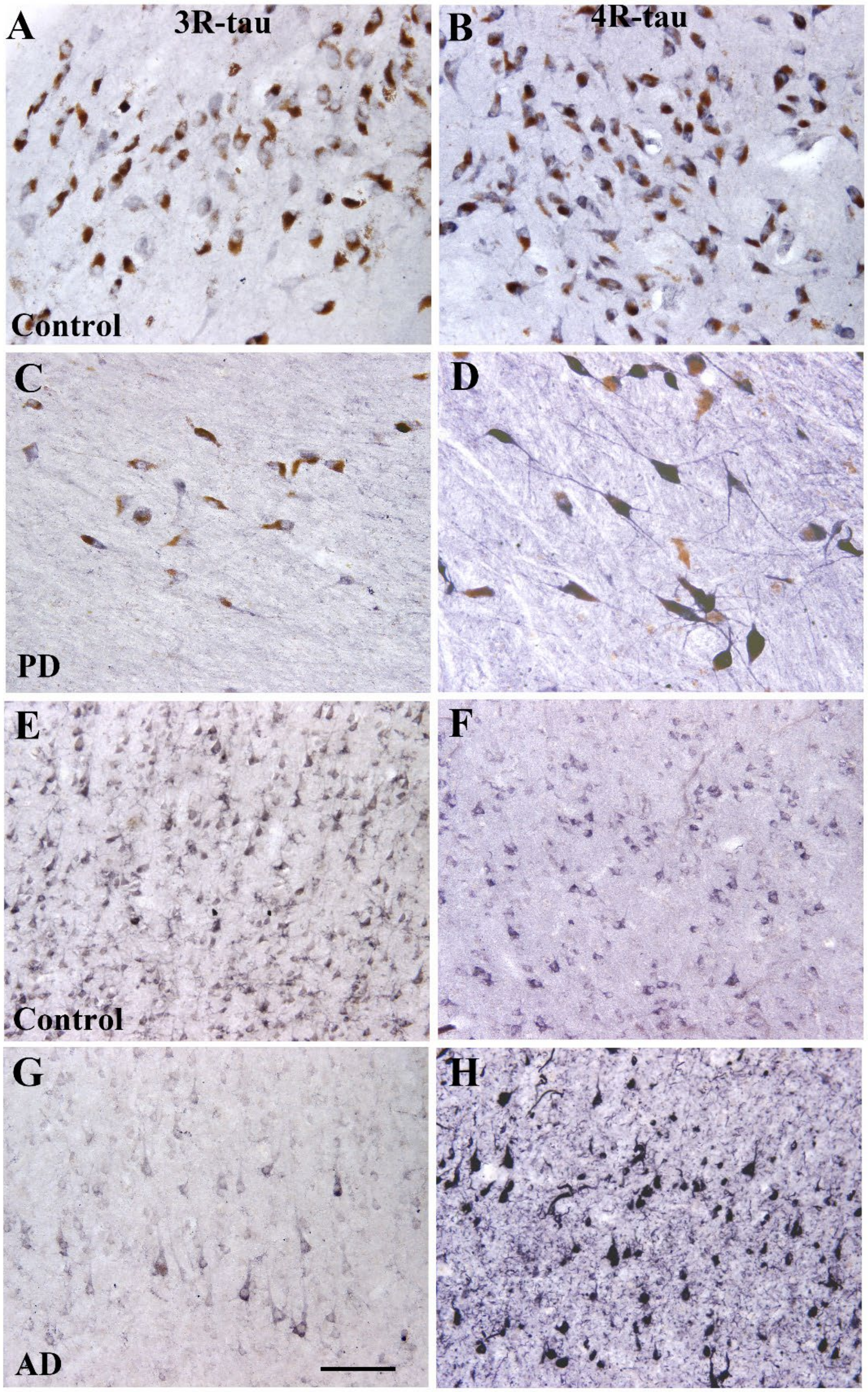

Supplementary Fig. 6. Comparison of 3R and 4R tau expression.

Photomicrographs of nigral (A-D) and temporal cortical (E-H) sections from subjects with NMD (age-matched controls, A, B, E, F), PD (C, D), and AD (G, H) illustrated 3R tau (Left column) and 4R tau (right column) immunostaining patterns. Note that both 3R (A, B) and 4R (E, F) tau immunolabeling displayed similar intensity in nigral and cortical neurons of age-matched controls. Light 3R (C, G) and intensive 4R (D, H) tau immunostaining were distributed in substantia nigra of PD and temporal cortex of AD. Scale bar in T =100 μm (applies to all).

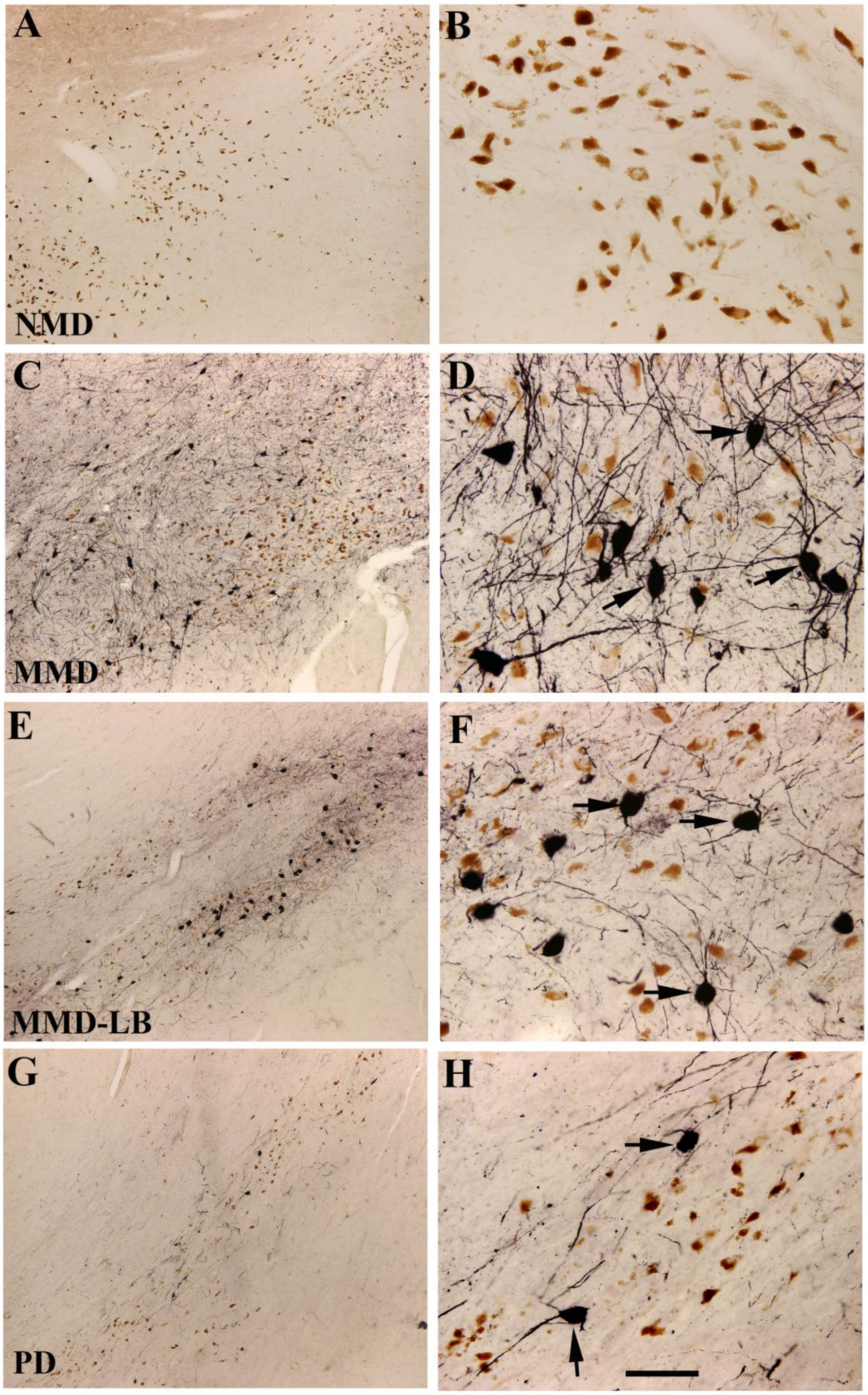

Supplementary Fig. 7. Qualitative observation of tau phosphorylated at serine-202 in substantia nigra. Photomicrographs of the mid-substantia nigra from no motor deficit (NMD; A, B), minimal motor deficits (MMD; C, D), minimal motor deficits with nigral Lewy body (MMD-LB; E, F), and Parkinson's disease (PD; G, H) show phosphorylated Serine-202 (CP13) tau patterns. CP13 immunoreactivity was undetectable in the subjects with NMD (A, B). In contrast, CP13-immunoreactive nigral neurons were observed in subjects with MMD (C, D), MMD-LB (E, F), and patients with PD (G, H). Note there were more CP13–immunoreactive neurons with extensive processes in subjects with MMD (arrows, D) and MMD-LB (arrows, F) than the patients with PD (arrows, H). Scale bar in H = 100 µm for B, D, F; 500 µm for A, C, E, G.

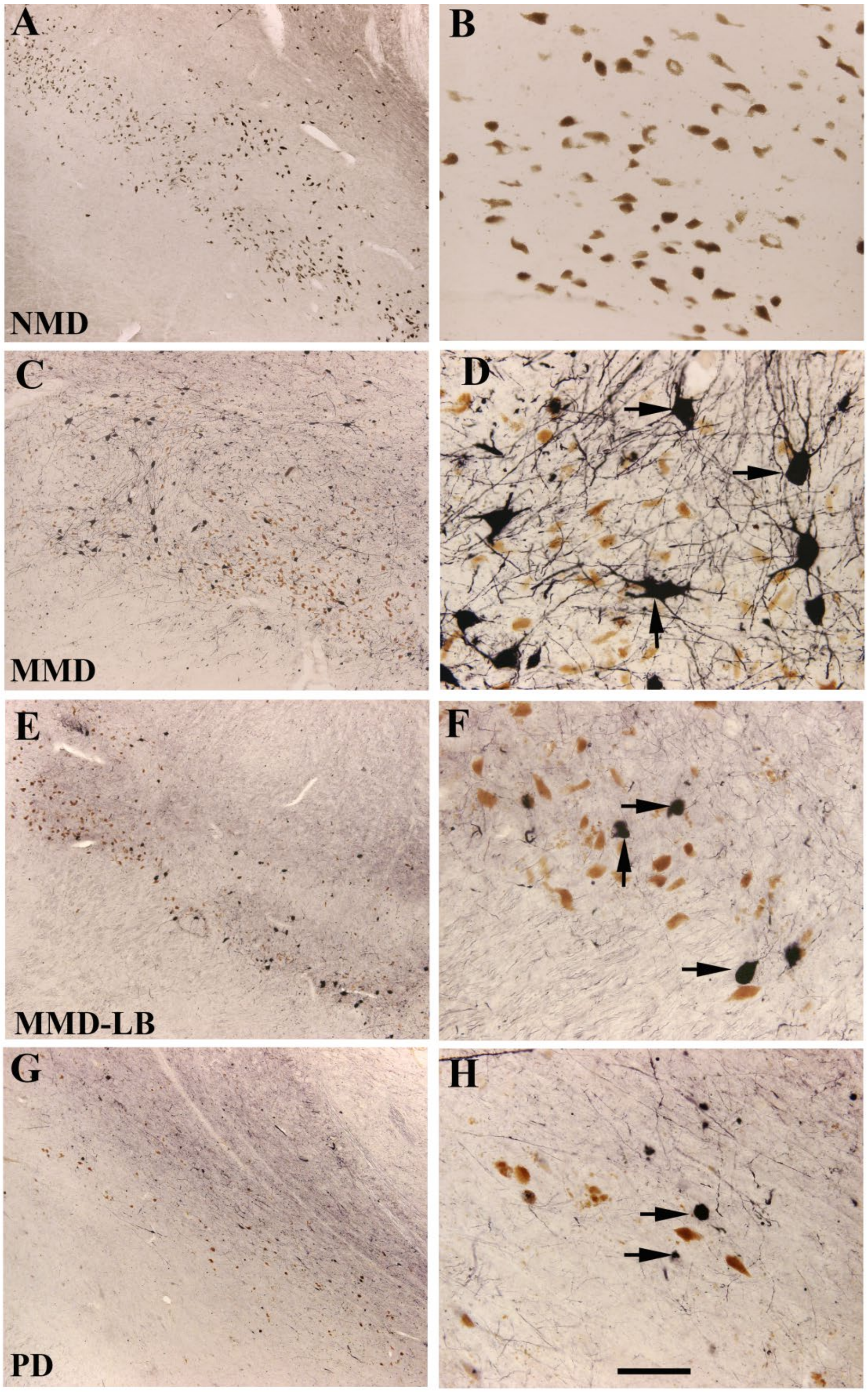

Supplementary Fig. 8. Qualitative observation of tau phosphorylated at Ser396/Ser404 in substantia nigra. Photomicrographs of the mid-substantia nigra from no motor deficit (NMD; A, B), minimal motor deficits (MMD; C, D), minimal motor deficits with nigral Lewy body (MMD-LB; E, F), and Parkinson's disease (PD; G, H) show phosphorylated Ser396/Ser404 (PHF-1) tau patterns. PHF-1 immunoreactivity was detected in the subjects with MMD (C, D), MMD-LB (E, F) and PD (G, H) but not NMD (A, B). Note that PFH-1–immunoreactive perikarya with extensive processes in subjects with MMD (arrows, D) while restricted processes in MMD-LB (arrows, F) and PD (arrows, H). Scale bar in H= 100 μm for B, D, F; 500 μm for A, C, E, G.

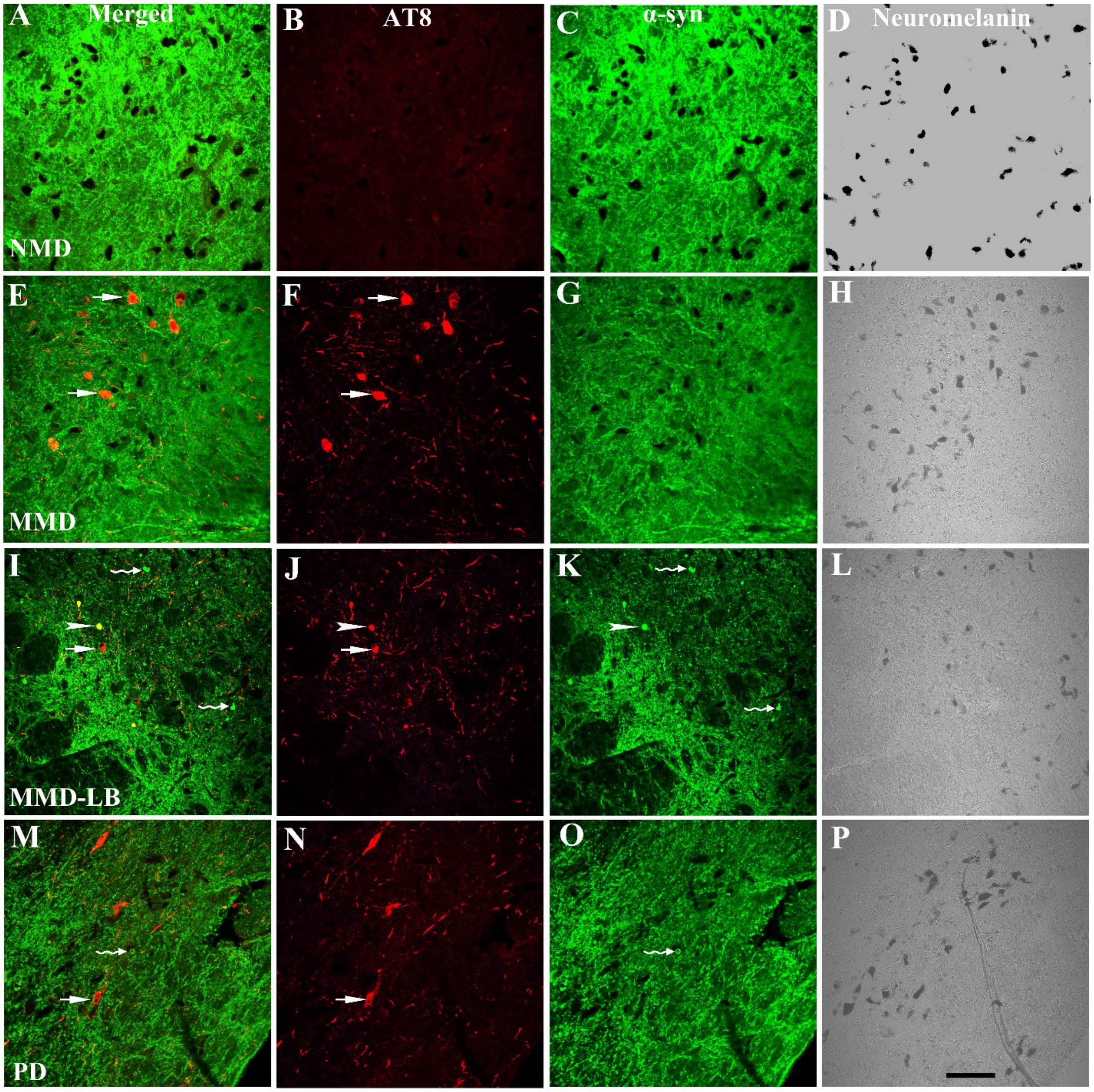

Supplementary Fig. 9. Co-localization analyses of phosphorylated tau (AT8) and endogenous levels of total α-synuclein in substantia nigra.

Confocal microscopic images of substantia nigra from no motor deficit (NMD; A-D), minimal motor deficits (MMD; E-H), minimal motor deficits with nigral Lewy body (MMD-LB; I-L) , and Parkinson's disease (PD; M-P) illustrated AT8 (red; B, F, J, N), α-synuclein (α-syn, green; C, G, K, O), neuromelanin (black; D, H, L, P) and co-localization of AT8 and α-synuclein (merged; A, E, I, M). Note no AT8 and α-syn aggregate in NMD (A-C), AT8 aggregates (arrows, E-G)but no α-syn aggregate in MMD, α-syn (curved arrow, I, K) and AT8 (arrow, I, J) aggregates deposited in nigral neurons separately and few AT8-labeling aggregates colocalized with α-syn (arrowhead, I, K) in MMD-LB, and α-syn (curved arrow, M, O) and AT8 (arrow, M, N) aggregates deposited in nigral neurons separately in PD. The α-syn-labeling fibers and dots were reduced in MMD (E, G), MMD-LB (I, K), and PD (M, O) as compared to NMD (A, C). Scale bar = 100 μm in P (applies to all).
